# Diurnal RNAPII-tethered chromatin interactions are associated with rhythmic gene expression in rice

**DOI:** 10.1186/s13059-021-02594-7

**Published:** 2022-01-06

**Authors:** Li Deng, Baibai Gao, Lun Zhao, Ying Zhang, Qing Zhang, Minrong Guo, Yongqing Yang, Shuangqi Wang, Liang Xie, Hao Lou, Meng Ma, Wei Zhang, Zhilin Cao, Qinghua Zhang, C. Robertson McClung, Guoliang Li, Xingwang Li

**Affiliations:** 1 National Key Laboratory of Crop Genetic Improvement, Hubei Hongshan Laboratory, Huazhong Agricultural University, 1 Shizishan Street, Hongshan District, Wuhan, 430070 Hubei China; 2 Department of Resources and Environment, Henan University of Engineering, 1 Xianghe Road, Longhu Town, Zhengzhou, 451191 Henan China; 3 Department of Biological Sciences, Dartmouth College, Hanover, NH 03755 USA; 4 Agricultural Bioinformatics Key Laboratory of Hubei Province and Hubei Engineering Technology Research Center of Agricultural Big Data, 3D Genomics Research Center, Huazhong Agricultural University, 1 Shizishan Street, Hongshan District, Wuhan, 430070 Hubei China

**Keywords:** RNAPII occupancy, Rhythmically expressed genes, Chromatin interactions, Chromatin spatial clusters, Chromatin connectivity networks

## Abstract

**Background:**

The daily cycling of plant physiological processes is speculated to arise from the coordinated rhythms of gene expression. However, the dynamics of diurnal 3D genome architecture and their potential functions underlying the rhythmic gene expression remain unclear.

**Results:**

Here, we reveal the genome-wide rhythmic occupancy of RNA polymerase II (RNAPII), which precedes mRNA accumulation by approximately 2 h. Rhythmic RNAPII binding dynamically correlates with RNAPII-mediated chromatin architecture remodeling at the genomic level of chromatin interactions, spatial clusters, and chromatin connectivity maps, which are associated with the circadian rhythm of gene expression. Rhythmically expressed genes within the same peak phases of expression are preferentially tethered by RNAPII for coordinated transcription. RNAPII-associated chromatin spatial clusters (CSCs) show high plasticity during the circadian cycle, and rhythmically expressed genes in the morning phase and non-rhythmically expressed genes in the evening phase tend to be enriched in RNAPII-associated CSCs to orchestrate expression. Core circadian clock genes are associated with RNAPII-mediated highly connected chromatin connectivity networks in the morning in contrast to the scattered, sporadic spatial chromatin connectivity in the evening; this indicates that they are transcribed within physical proximity to each other during the AM circadian window and are located in discrete “transcriptional factory” foci in the evening, linking chromatin architecture to coordinated transcription outputs.

**Conclusion:**

Our findings uncover fundamental diurnal genome folding principles in plants and reveal a distinct higher-order chromosome organization that is crucial for coordinating diurnal dynamics of transcriptional regulation.

**Supplementary Information:**

The online version contains supplementary material available at 10.1186/s13059-021-02594-7.

## Background

Diurnal oscillations of gene expression are presumed to drive daily cycles of plant physiological processes through core circadian genes involved in interlocked transcriptional/translational negative feedback loops [1–8]. The genome-wide *cis*-acting targets (cistromes) of core circadian clock components during the circadian cycle have been identified in mammals using the high-throughput chromatin immunoprecipitation (ChIP) approach [9–11]. Interlocking transcriptional loops generate cycles of transcription with various expression phases depending on the alternative usage of *cis*-elements in the promoters and enhancers of specific target genes [12, 13]. In *Arabidopsis*, genome-wide identification of circadian clock associated 1 (*CCA1*), the timing of CAB expression 1 (*TOC1*; also known as *PRR1*), pseudo-response regulator 5 (*PRR5*), and *PRR7* targets revealed phase-specific circadian clock regulatory elements in target gene promoters and temporal regulation of specific output pathways [14–17].

In the transcriptional feedback loop module, chromatin status and RNA polymerase II (RNAPII) occupancy are directly linked to the regulation of diurnal gene expression patterns [6, 8, 13]. In the mouse liver, on a genome-wide level, circadian rhythms in transcription factor (TF) binding, RNAPII recruitment and initiation, and chromatin states account for cycling mRNA transcripts, suggesting that RNAPII occupancy and histone modifications are intimately connected with the generation of diurnal output rhythms [11, 13]. Accumulating evidence has shown that chromatin remodeling events are involved in circadian regulation in plants. In *Arabidopsis*, core clock proteins interact with epigenetic modifiers, and the related chromatin-modifying enzymes are associated with promoters bearing rhythmic mark deposition [18–21]. In addition, core circadian clock genes promote diurnal expression of chromatin-modifying factors, which in turn regulate rhythmic histone modification on the core clock and target genes [22]. RNAPII plays an essential role in initiating nascent transcription [23, 24]; however, whether RNAPII occupancy is under diurnal control to regulate circadian transcription in plants remains poorly understood.

Over the past few years, chromatin conformation capture techniques have provided an unprecedented three-dimensional (3D) view of chromatin organization [25, 26]. Recent advances in 3D genome studies have shown that long-range chromatin interactions between promoters and enhancers influence the expression of connected genes in a coordinated pattern [27–30]. In addition, recent studies have described the circadian regulation underlying genome-wide higher-order genome organization and long-range interactions at specific loci in mammals [31–36]. However, despite this progress, a comprehensive understanding of the high-resolution dynamic chromatin architecture and its effects on diurnal gene expression is still lacking in plants.

To investigate how RNAPII dynamically interacts with their corresponding regulatory regions during the circadian cycle, we used long-read chromatin interaction analysis (ChIA-PET) to globally analyze genome-wide chromatin interactions associated with RNAPII in rice. We also systematically characterized the interplay between 3D genome architecture, rhythmic RNAPII occupancy, and rhythmic gene expression. The generated RNAPII-associated high-resolution dynamic chromatin interactome maps revealed a detailed chromatin topology that provides a framework for understanding orchestrated circadian transcriptional outputs.

## Results

### RNAPII occupancy is rhythmic and predicts transcription in rice

**Fig. 1:**
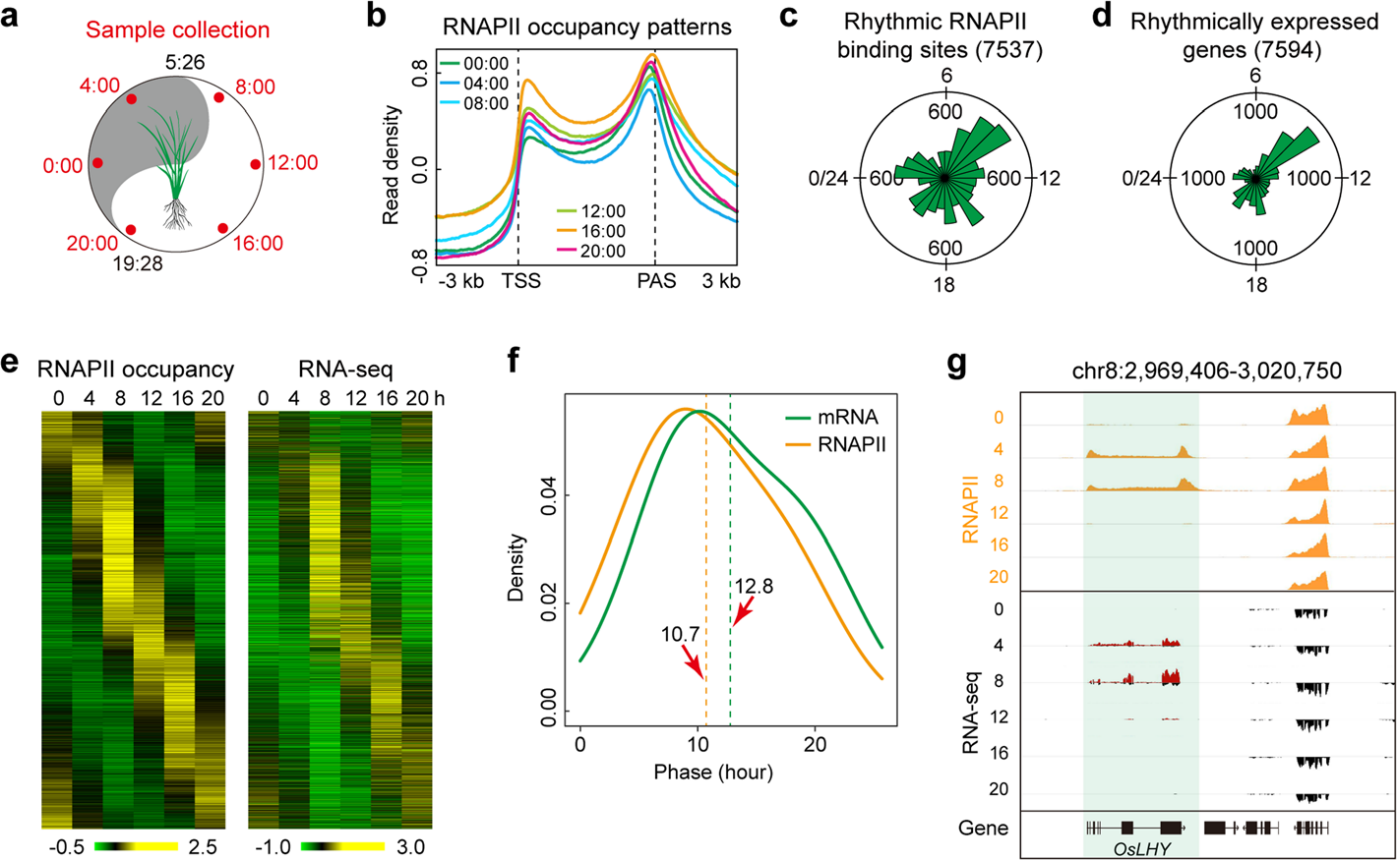
Rhythmic occupancy of RNAPII and temporal relationship with mRNA accumulation in rice. **a** Sampling time points of a diurnal light and dark cycle under field conditions. Red font indicates the sample collection time, and black font indicates sunrise and sunset. **b** Distribution of RNAPII occupancy along genes over a six-point time course in mature leaves of rice. The gene body was converted into percentiles to standardize genes with different lengths. Regions 3 kb upstream and downstream of the gene are shown. **c** Phase distribution of rhythmic RNAPII occupancy represented as a rose plot. The values inside the circle represent the coordinates, the numbers outside the circle indicate the sidereal hours. **d** Peak expression phase distribution of rhythmically expressed genes. The phase of each transcript rhythm is represented as a rose plot. **e** Heat map representation of rhythmic RNAPII occupancy and transcript levels. Each rhythmic RNAPII occupancy and the corresponding rhythmically expressed gene are represented as a horizontal line and ordered vertically by the rhythmic RNAPII occupancy phase in sidereal hours. **f** Density plot of RNAPII occupancy and mRNA accumulation phase. The orange dashed line indicates the average phase of RNAPII occupancy, and the green dashed line indicates the average phase of mRNA accumulation. **g** Features of RNAPII binding peaks and gene transcription at the indicated time point and the *OsLHY* locus. Each RNAPII occupancy and RNA-seq track represents the normalized read coverage (wiggle plot) at a single time point. Of the RNA-seq track, the red and black wiggle plots represent forward- and reverse-strand RNA-seq reads, respectively. Six time points (each time point is a replicate of two successive days) every 4 h over a circadian cycle are shown beginning at 00:00 and ending at 20:00

To explore the origin of the global rhythms of gene expression in rice, we analyzed the genome-wide occupancy of RNAPII using ChIP-seq over a six-point time course spanning 24 h (Fig. 1a, Fig. S1, Additional file 2: Table S1). We found that RNAPII signals increased sharply at transcription start sites (TSS), decreased on entering gene body regions, and peaked at polyadenylation site (PAS) (Fig. 1b). On a genome-wide basis, we detected 21,495 RNAPII-binding peaks and found that 35% (7537) of RNAPII occupancy was rhythmic (Fig. S2a, Additional file 3: Table S2). We also detected 7594 rhythmically expressed genes (37%, out of 20,696 active genes) as determined by BIO_CYCLE [37] [*q* < 0.05, 20 < period (*t*) < 28 h] (Fig. S3a, Additional file 4: Table S3 and Additional file 5: Table S4). We then examined the temporal relationships between RNAPII recruitment and RNA accumulation and found that the phase distribution of RNAPII peaks and peak abundance of rhythmic transcripts clustered around the morning phase (Fig. 1c, d; Fig. S2b, 3b) and that rhythmic RNAPII peak intensity was significantly correlated with rhythmic transcript expression levels (Pearson rank correlation = 0.69, over the six time points; Fig. 1e, Fig. S2c). In addition, a global analysis of phase relationships revealed that RNAPII recruitment preceded RNA accumulation by 2 h (Fig. 1f, g). However, the open chromatin regions were non-rhythmic over the sampled time series (Fig. S2a). The rhythmic mRNA expression of known circadian-related genes (e.g., *OsLHY*, *OsTOC1*) showed robust oscillation patterns (Fig. S3c). The Kyoto Encyclopedia of Genes and Genomes (KEGG) pathway analysis indicated that genes oscillating in phase have closely related biological functions (Fig. S3d) that vary with the phase, supporting the robustness of our data. Together, these results suggest that genome-wide rhythmic RNAPII recruitment largely influences the oscillating transcriptome and that widespread transcriptional and post-transcriptional regulatory events also contribute to the generation of rhythmic mRNA in rice.

### Rhythmic RNAPII recruitment is associated with diurnal 3D genome architecture remodeling

**Fig. 2:**
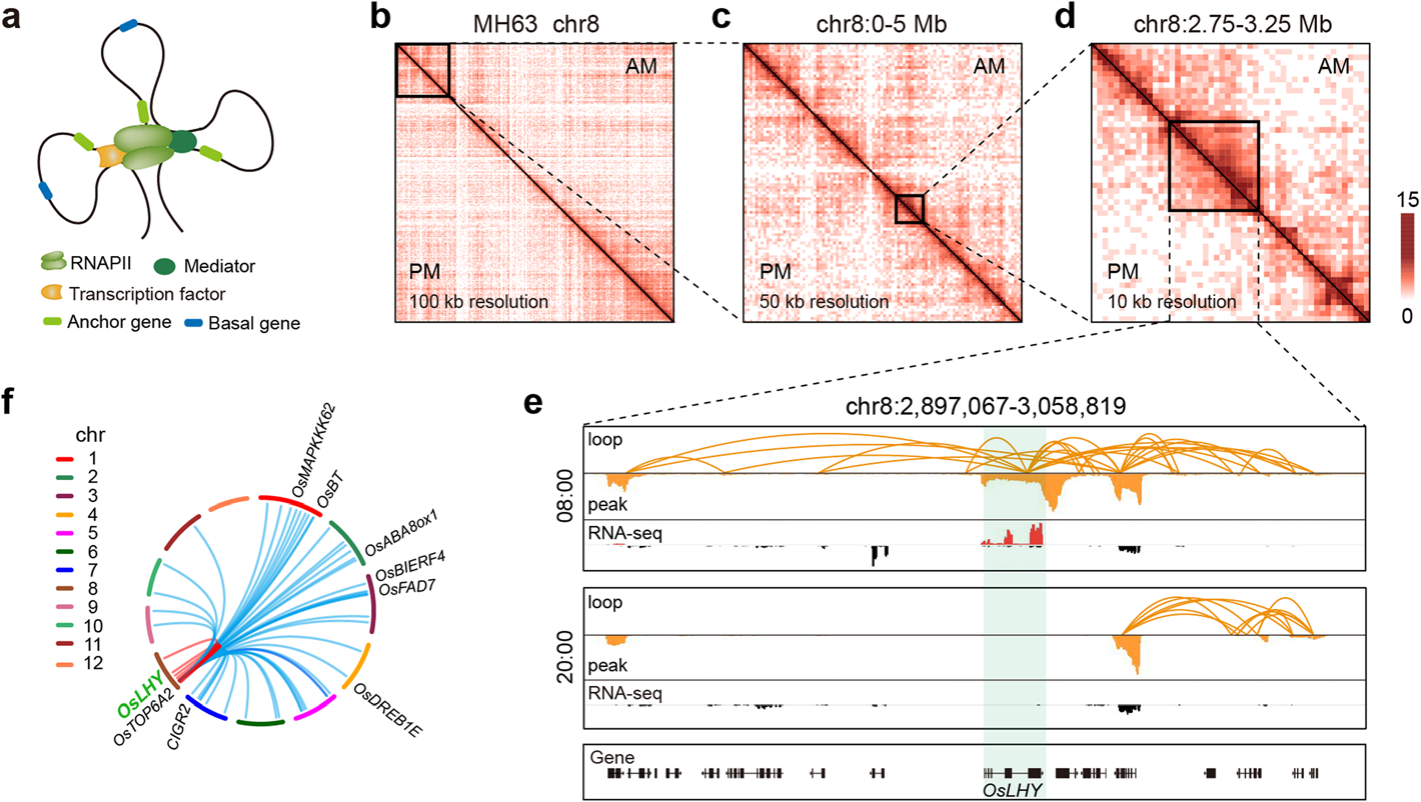
ChIA-PET analysis defines the RNAPII interactome in rice during a circadian cycle. **a** ChIA-PET mapping of the long-range chromatin interactions of genes. **b–d** ChIA-PET contact heatmaps at 100, 50, and 10 kb resolution of the 08:00 (AM) and 20:00 (PM) datasets. **e** Mapping browser screenshot showing the RNAPII-defined chromatin interactions, RNAPII occupancy, and RNA-seq data in the box region of **d**. Each chromatin interaction, RNAPII occupancy, and RNA-seq track represents the normalized loops and read coverage (wiggle plot) at the 08:00 and 20:00 time points. **f** Circos plot representing the genome-wide view of *OsLHY* interactions with the corresponding chromosomes in *trans* at 08:00. The cloned gene corresponding to each contact region is indicated in the outer layer of the plot. The lines in red represent intrachromosomal interactions, and lines in blue represent interchromosomal interactions

**Fig. 3:**
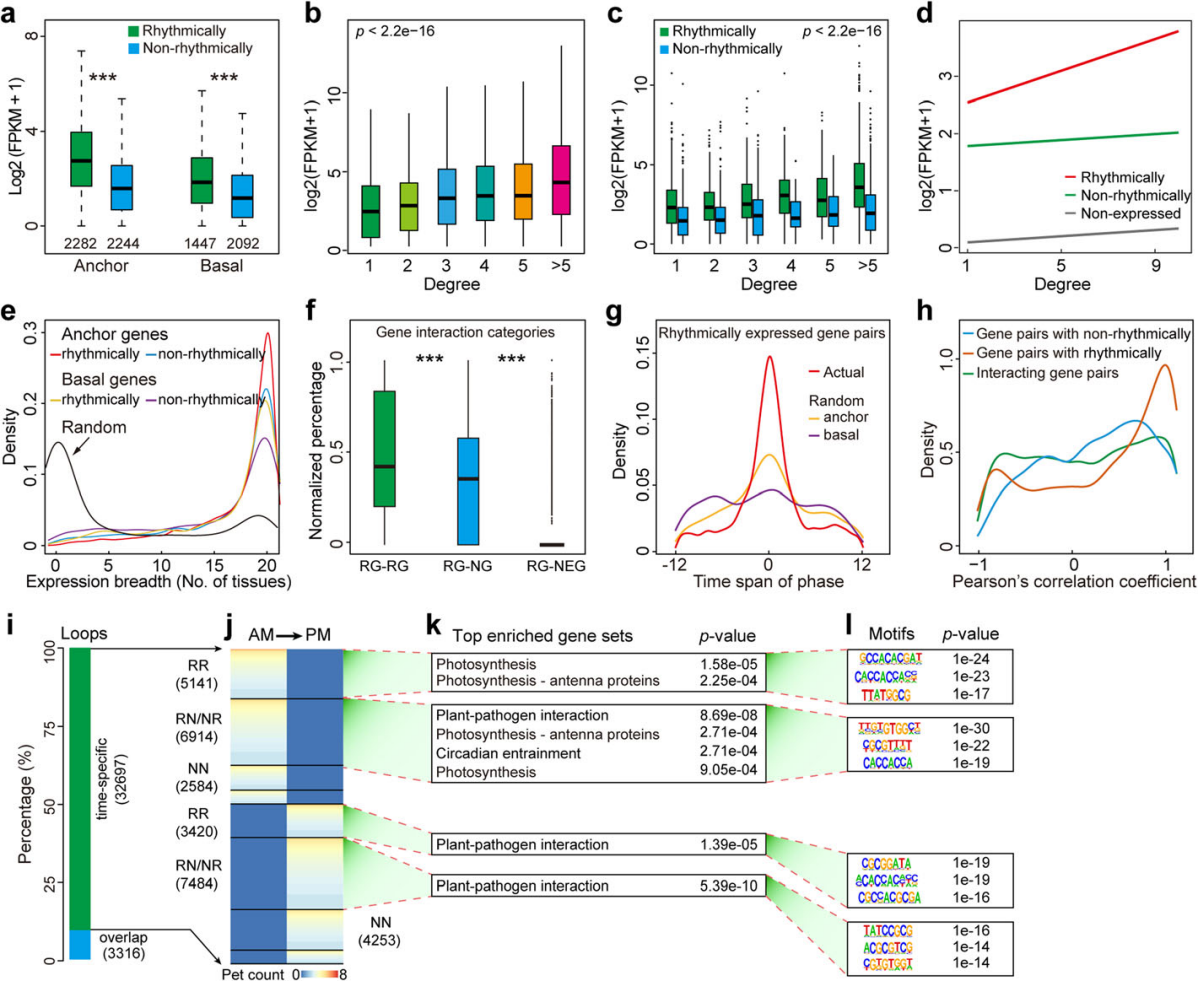
Characterization of rhythmically expressed gene-centered chromatin interactions and dynamics of chromatin loops. **a** Boxplot showing the expression levels of rhythmically and non-rhythmically expressed genes involved or not involved in RNAPII-mediated chromatin interactions at 08:00. ***p < 0.0001. **b** Boxplot showing the positive correlation between the degrees (connection frequency) and transcript abundance of anchor genes at 08:00. **c** Boxplot showing the positive relationship between the degrees and transcript abundance of rhythmically and non-rhythmically expressed anchor genes at 08:00. **d** As the degree increases, rhythmically expressed anchor genes showed higher transcript abundance than did non-rhythmically expressed anchor genes did at 08:00. The correlation coefficients between transcript abundance and degree among rhythmically, non-rhythmically, and non-expressed genes were 0.94, 0.42, and 0.51, respectively. Rhythmically vs. non-rhythmically, *p* = 9.669e−06 (binomial test). **e** Expression breadth (number of tissues in which a gene is expressed) of RNAPII-mediated anchor and basal genes with rhythmic or non-rhythmic characteristics. Random genes served as controls. **f** Distribution of rhythmically expressed gene-centric interactions at 08:00. The percentages of RG (rhythmically expressed gene)-RG, RG-NG (non-rhythmically expressed gene), and RG-NEG (non-expressed gene) interactions are listed. ***p < 0.0001. **g** Distribution of phase spans of RG-RG interaction gene pairs and randomly picked gene pairs from rhythmically expressed anchor genes and rhythmically expressed basal genes in the 08:00 datasets. Actual vs. random anchor, *p* = 6.469e−11; actual vs. random basal, *p* < 2.2e−16, random anchor vs. random basal, *p* = 1.057e−07 (Kolmogorov-Smirnov test). **h** Distribution of Pearson’s correlation coefficient for RNAPII-bound interacting gene pairs, gene pairs with a loop connecting rhythmically expressed genes, and gene pairs with a loop connecting non-rhythmically expressed genes in the 08:00 datasets. **i** Bar chart showing the percentages of overlap (common) and dynamic chromatin loops across the day. **j** Heatmap showing the time-specific chromatin loops, ordered by the number of paired-end tag (PET) counts. Pseudo-color reflects the normalized contact frequencies between the loop anchors for each stage-specific loop. RR, RG-RG; RN/NR, RG-NG or NG-RG; NN, and NG-NG. **k** Enriched KEGG pathways for genes located at the loop anchors of distinct clusters in **j** along with their adjusted *p* values. **l** Over-represented transcription factor-binding motifs for distal open chromatin regions located at the loop anchors of distinct clusters in **j** and their corresponding *p* values

Rhythmic RNAPII occupancy prompted us to examine whether the RNAPII-mediated high-resolution 3D genome organization changes in a rhythmic manner. We thus performed RNAPII ChIA-PET using the same samples employed for ChIP-seq and RNA-seq at 08:00 and 20:00 (Fig. 2a, Fig. S4a, b, Additional file 6: Table S5). In total, we identified 20,667 high-confidence (FDR < 0.05) RNAPII-associated chromatin interactions connecting 4526 genes at 08:00 (Fig. 3a, Additional file 7: Table S6) and 21,001 loops connecting 5298 genes at 20:00 (Fig. S6e, Additional file 8: Table S7). Approximately half (48.63%) of these loops were intrachromosomal and unevenly distributed along the genome (Fig. S5). The genomic spans of the 08:00-specific intrachromosomal loops were longer than those of the 20:00-specific intrachromosomal loops and common intrachromosomal loops (Fig. S4c). The overall megabase-sized RNAPII-associated 3D genome organization was highly similar between 08:00 and 20:00. However, visualization of progressively enlarged genomic regions in the genome browser with chromatin interactions showed a clear divergence between 08:00 and 20:00 (Fig. 2b–d). *OsLHY*, a well-known core circadian clock gene in rice [38], was found to form loops with 74 genomic regions at 08:00 in the RNAPII-mediated chromatin connectivity maps (Fig. 2e, f, Additional file 9: Table S8), which is the time point at which *OsLHY* showed the highest expression level. In contrast, these RNAPII-tethered chromatin loops were virtually undetectable at 20:00 at *OsLHY* locus, which was the time point of trough *OsLHY* expression (Fig. 2e). These findings demonstrate that RNAPII-related rhythmic spatial clustering at the *OsLHY* locus is highly time-specific and that these variations in looping structures may influence the diurnal expression of *OsLHY*.

### Properties of rhythmically expressed genes involved in the RNAPII-mediated diurnal 3D genome architecture

Among the 13,738 and 16,983 RNAPII-binding sites detected at 08:00 and 20:00, respectively, 8455 peaks were overlapped. Of 6090 (44%) and 6883 (41%) chromatin interaction anchors at 08:00 and 20:00, respectively, we found 3608 shared RNAPII anchors between two time points. The remaining RNAPII peaks were classified as basal promoters without chromatin interactions (Fig. S6a, b). The peak intensity of the anchor sites was significantly higher than that of the basal sites, and the intensity of rhythmic peaks was significantly higher than that of non-rhythmic peaks in the corresponding anchor or basal sites, suggesting that rhythmic RNAPII peaks with higher peak intensities are more likely to be involved in chromatin loops (Fig. S6a–d). Moreover, the transcript abundance of both rhythmically expressed anchor and basal genes was significantly higher than that of non-rhythmically expressed genes in the corresponding models (Fig. 3a, Fig. S6e). In addition, anchor genes with high degrees (connection frequency) displayed high expression levels, and rhythmically expressed anchor genes showed greater transcript abundance than non-rhythmically expressed anchor genes in the AM datasets (Fig. 3b–d). These trends were also present in the PM datasets, although the differences between the rhythmically and non-rhythmically expressed anchor genes were less pronounced (Fig. S6f–h). Gene expression breadth analysis (breadth is defined as the number of tissues in which a transcript is detected) showed that anchor genes were more widely expressed than basal genes were, and further analysis revealed that rhythmically expressed anchor genes were almost universally expressed genes, supporting the notion that RNAPII-interacting anchor genes are specifically enriched for ubiquitously expressed genes (Fig. 3e, Fig. S6i, Additional file 10: Table S9).

Further analyses showed that ~ 74% of RNAPII-bound loops were associated with rhythmically expressed genes and that the rhythmically expressed gene pairs were more likely to be tethered by RNAPII than were rhythmically-non-rhythmically expressed gene pairs (Fig. 3f, Fig. S6j). Moreover, interacting rhythmically expressed gene pairs tended to have the same or close diurnal phase (Fig. 3g, Fig. S6k) and showed significantly higher positive transcriptional correlation compared with other categories (Fig. 3h, Fig. S6l). For example, of all the *OsLHY* looping genes, 46% (33), 44% (31), and 10% (7) were associated with rhythmically, non-rhythmically, and non-expressed genes, respectively, and the expression peak phases of rhythmically expressed genes were largely (70%, 23 out of 33) clustered in the 06:00–10:00 temporal window (Fig. S6m, n). The expression levels of RNAPII-mediated *OsLHY* looping rhythmic genes were positively correlated with *OsLHY* mRNA expression (Pearson rank correlation = 0.71), while other looping genes (non-rhythmically and non-expressed genes) were negatively correlated with *OsLHY* mRNA expression (Pearson rank correlation = − 0.38) (Fig. S6o). Altogether, our results indicate that rhythmically expressed genes showing small phase differences are more likely to be involved in RNAPII-mediated organization for coordinated transcription.

More than half (58%; 2447) of the rhythmic loop anchors were time-specific, and the peak intensity was significantly correlated with the level of rhythmic transcripts (Pearson rank correlation = 0.79; Fig. S7a, b). Moreover, 91% of the chromatin interactions (32,697) were time-specific (Fig. 3i). Actually, of all the 32,697 time-specific chromatin loops, ~ 26% (*n* = 8561) were RG (rhythmically expressed genes)-RG interactions and looped 2619 rhythmically expressed genes; ~ 44% (*n* = 14,398) were RG–NG (non-rhythmically expressed genes) or NG–RG interactions and looped 4757 rhythmically expressed genes and 2383 non-rhythmically expressed genes, respectively; ~ 21% (*n* = 6,837) were NG–NG interactions and looped 2280 non-rhythmically expressed genes; and ~ 9% (*n* = 2901) were NEG (non-expressed genes)-associated interactions, and of all the looping genes, 37% (780), 31% (656), and 32% (694), were associated with rhythmically, non-rhythmically, and non-expressed genes, respectively (Fig. S7c). In total, there are 3259 rhythmically (43%, out of 7594 rhythmically expressed genes), 3241 non-rhythmically (25%, out of 13,102 non-rhythmically expressed genes), and 694 non-expressed (2%, out of 36,478 non-expressed genes) genes involved in RNAPII-associated time-specific chromatin interactions. We also defined two distinct clusters of time-specific chromatin loops, each subdivided by the corresponding rhythmic characteristics of anchor genes (Fig. 3j). In particular, the gene set engaged in the RNAPII-associated AM chromatin loop cluster was enriched for biological processes, including photosynthesis and circadian entrainment, whereas the gene set involved in the PM chromatin loop cluster was enriched for plant–pathogen interactions (Fig. 3k, l), demonstrating that gene clusters related to altered chromatin loops participating in diverse biological pathways.

### RNAPII-associated chromatin spatial clusters (CSCs) change throughout the day, and variable CSCs are correlated with gene expression

**Fig. 4:**
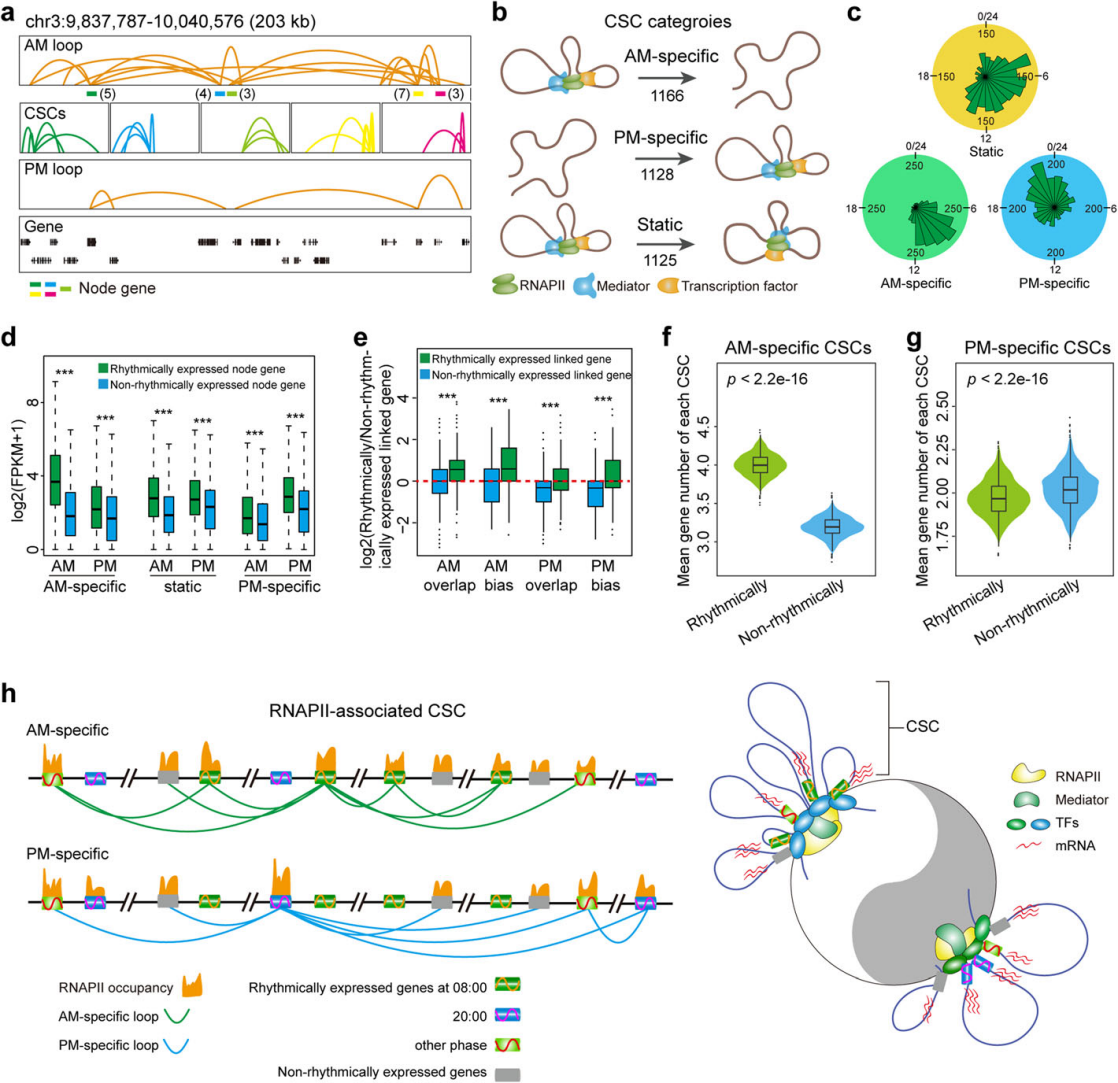
RNAPII-organized chromatin spatial clusters (CSCs) change throughout the day. **a** Browser view of a 203-kb genomic segment with five CSCs showing RNAPII ChIA-PET data at the 08:00 (AM) and 20:00 (PM) time points. The number in brackets represents the number of connecting genes in each CSC. **b** Simulated 3D model of the CSCs throughout the diurnal cycle. CSCs change in an oscillatory manner with three forms: (i) an AM-specific state, (ii) a PM-specific state, and (iii) a static state (overlap). **c** The phase distribution of rhythmically expressed node genes in the three possible models specified in **b** represented as rose plots. **d** Expression levels of rhythmically or non-rhythmically expressed node genes in the three possible models. ***p < 0.0001. **e** Boxplot shows that rhythmically expressed node genes within AM CSCs tended to connect with rhythmically expressed genes, whereas non-rhythmically expressed node genes in PM CSCs tended to connect with non-rhythmically expressed genes. The red dashed line indicates the number of rhythmically expressed linked genes equally to the number of non-rhythmically expressed linked genes within a CSC. ***p < 0.0001. **f**, **g** Violin plot showing the distribution of the mean gene number in AM-specific CSCs (**f**) and PM-specific CSCs (**g**) with randomly simulated rhythmically and non-rhythmically expressed genes. **h** Schematic model of the chromatin organization during the circadian cycle. Representative CSCs from AM and PM circadian windows as well as their components and influence on rhythmically and non-rhythmically expressed genes transcription are shown. Red wavy lines indicate transcript abundance.

RNAPII-mediated chromatin loops were observed to form spatial clusters, in which multiple interactions could be seen emanating from a single anchor site termed the node gene; the divergent interaction sites were considered the connecting genes (Fig. 4a). We found that these CSCs showed considerable variation between the 08:00 and 20:00 datasets that reflected three types of oscillatory patterns: (i) a total of 1166 AM-specific CSCs were identified at 08:00 that were absent or markedly decreased in size at 20:00 as the diurnal cycle progressed. (ii) a total of 1128 PM-specific CSCs were present at 20:00 that were absent or markedly decreased in size at 08:00, and (iii) a total of 1125 static CSCs were present at both 08:00 and 20:00, which included common node genes with different connecting genes in the morning and evening (Fig. 4b).

In all three types of CSC examined, approximately two-thirds of the node genes were associated with rhythmically expressed genes, 20–40% of the node genes were associated with non-rhythmically expressed genes, and the rest were not expressed (Fig. S8a); moreover, the percentages were varied with the type of CSC. Rhythmically expressed node genes within AM-specific CSCs were enriched during their expression phase at 08:00 and showed higher expression levels than at 20:00 (Fig. 4c, d, Fig. S8a). Similarly, node genes in PM-specific CSCs were enriched with rhythmically expressed genes in the evening phase and showed higher levels of gene expression at 20:00 than at 08:00 (Fig. 4c, d, Fig. S8a). Rhythmically expressed node genes within static CSCs with expression peaks were relatively randomly distributed throughout the day and had similar expression levels in the morning and evening (Fig. 4c, d, Fig. S8a). The non-rhythmically expressed node genes in each category showed lower expression levels but similar expression patterns to the rhythmically expressed node genes (Fig. 4c, d, Fig. S8a). In addition, rhythmically expressed node genes in the AM-specific CSCs had higher expression levels (Fig. S8b) and degrees (Fig. S8c), stronger correlation of expression (Fig. S8d), and a more concentrated phase difference (Fig. S8e) than those in PM-specific CSCs. This reflects a diverse RNAPII-associated chromosomal organization associated with rhythmically expressed genes in the AM circadian phase compared with those in the PM phase. Furthermore, rhythmically expressed node genes within AM CSCs were preferentially linked to rhythmically expressed genes as their linked genes, whereas non-rhythmically expressed node genes in PM CSCs were preferentially linked to non-rhythmically expressed genes (Fig. 4e). The findings suggest that RNAPII preferentially capture rhythmically expressed genes in the morning and non-rhythmically expressed genes in the evening to form spatial clusters.

Static CSCs shared common node genes but had distinctive connecting genes between the AM and PM datasets. Notably, the common node genes shared approximately 10% common linked genes and possessed 90% AM- or PM-specific linked genes (Fig. S8f, g). These results indicate that node gene-centric CSCs may connect distinct clock components in the interconnected circuits between morning and evening.

We further examined whether RNAPII-associated time-specific CSCs underlie the genome architecture facilitating cooperative gene expression. Compared with non-rhythmically expressed genes, rhythmically expressed genes tended to be enriched in AM CSCs (including AM-specific and common CSCs) (Fig. 4f, Fig. S8h). Peak phase analysis revealed that of the 2337 rhythmically expressed genes that peaked at 08:00, the actual number of genes involved in each AM-specific CSC was 4.87, which was far more than that for the same number of randomly simulated genes (Fig. 4f). This suggests that phase-related genes tend to form spatial clusters, and such spatial congregation may contribute to the coordinated expression of rhythmically expressed genes in the morning. In contrast, non-rhythmically expressed genes were prone to tether together in PM CSCs (Fig. 4g, Fig. S8i), which was consistent with the evidence mentioned above (Fig. 4e). Together, these results suggest that RNAPII is more likely to tether rhythmically expressed genes in the AM phase and non-rhythmically expressed genes in the PM phase, linking chromatin architecture to orchestrated gene expression.

The above findings helped establish a 3D genome architecture model linked to transcription control (Fig. 4h). The model describes RNAPII-associated CSCs under diurnal control and spatial partitioning of the genome organization into CSCs that correlate with transcription. Specifically, RNAPII preferentially tethered rhythmically expressed genes in the morning, forming a hub for coordinated rhythmic expression. As the circadian cycle progresses, RNAPII-associated morning spatial clusters disassembled, and then RNAPII tended to capture non-rhythmically expressed genes together in the evening to orchestrate expression. Overall, RNAPII-associated genome-wide chromatin folding showed high plasticity during the circadian cycle, and the variable spatial clusters were correlated with transcription outputs.

### Complex dynamic patterns of chromatin interactome maps highlight an AM–PM switch of RNAPII-associated genome conformation

We further explored the global oscillations of the RNAPII-mediated high-resolution 3D chromatin organization during the morning and evening. We investigated the interaction frequencies of 50 genes upstream and downstream the TSS of node genes (degree ≥ 3), centering the interaction submatrices at the TSS while considering the transcription directionality. Notably, AM-specific node genes showed strong preferential clustering around the TSS in the AM datasets (Fig. S9a). Moreover, AM-specific node gene-associated interaction profiles in the PM datasets showed weak spatial clustering around the TSS (Fig. S9b). In contrast, PM-specific node genes displayed a temporally opposite change, from enhanced interactions around the TSS in PM datasets (Fig. S9c) to an overall reduction in interaction frequencies in the AM datasets (Fig. S9d). Together, these results indicate that the RNAPII interaction sites mapped by our ChIA-PET data dynamically change during the circadian cycle.

We then classified the core node genes (degree ≥ 32) located in the RNAPII-arranged networks into three groups based on the circadian patterns to explore their phase features (Fig. S10a). AM-unique core node genes had a higher amplitude, more concentrated phase distribution, and narrower expression breadth compared with that of PM-unique and overlapping core node genes (Fig. S10b–e), further suggesting that the RNAPII interaction maps in the AM may have a distinct nuclear landscape from that of the PM.

**Fig. 5:**
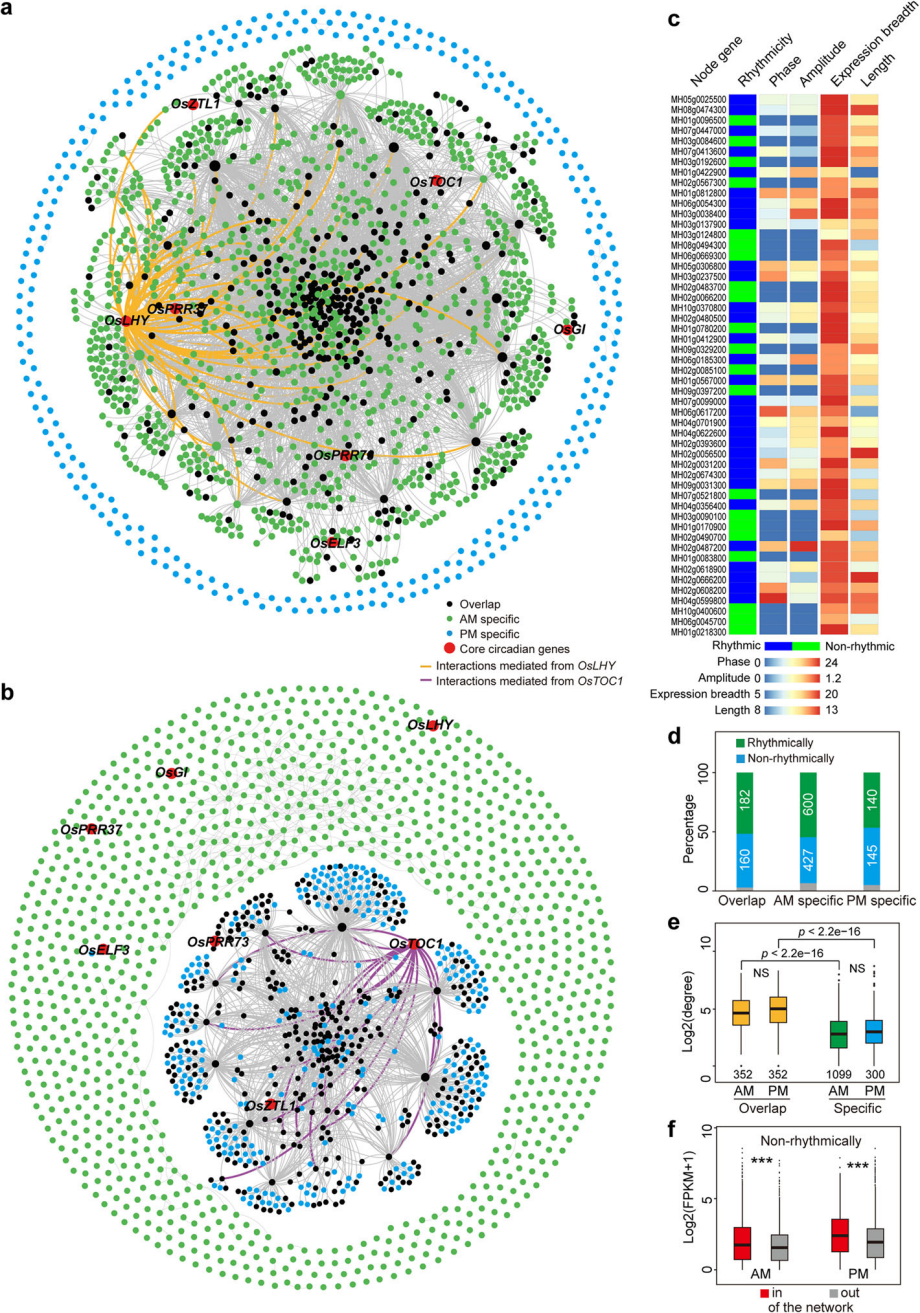
Connecting networks converged by core circadian genes. **a**, **b** Connectivity network constructed from two-hop interactions mediated from the core circadian genes at 08:00 (**a**) and 20:00 (**b**). The connectivity network was constructed through two hops of all interactions (light grey lines) mediated from seven (top; *OsLHY*, *OsTOC1*, *OsPRR73*, *OsPRR37*, *OsGI*, *OsZTL1*, and *OsELF3*) or three (bottom; *OsTOC1*, *OsPRR73*, and *OsZTL1*) genes. The colors of the circles represent the node features. Colored lines represent *OsLHY-*centric (top; yellow) or *OsTOC1-*centric (bottom; purple) interactions. The different node sizes represent the core circadian factor (large) versus the interaction factor (small) nodes. **c** Properties of the top 52 core node genes with the highest chromatin connectivity in the core circadian gene-associated chromatin connectivity maps. **d** Distribution of rhythmically and non-rhythmically expressed genes that participated in the core circadian gene-associated chromatin connectivity maps. Gene numbers in each category are given. **e** Boxplot showing the degrees of the common and specific chromatin connectivity genes in the networks. NS, no significant difference. **f** Boxplot showing the expression levels of non-rhythmically expressed genes involved or not involved in the core circadian gene-associated chromatin connectivity maps at 08:00 and 20:00. ***p < 0.0001

The global circadian transcriptome is driven by core circadian genes involved in autoregulatory loops [13, 39]. To investigate the spatial chromatin interaction network of the core circadian genes, we constructed interaction networks. We found that the core circadian gene regulatory landscape showed fundamentally different chromatin configurations between the AM and PM, as determined by subtraction of the 08:00 and 20:00 RNAPII-associated connectivity networks (Additional file 9: Table S8 and Additional file 11: Table S10). We were able to recapitulate the AM-specific interactions between *OsLHY* and the functionally related *OsTOC1*, *OsPRR73*, and *OsELF3* genes as well as the PM-specific disconnection of these loci (Fig. S11a, b). When network analysis was extended from one to two hops of connectivity, all core circadian genes were found to be connected within one major network (Fig. 5a, Fig. S11c). In contrast to the AM-specific connectivity networks, we observed a transition in the connectivity maps used to assess the loci of the core circadian genes; they exhibited scattered, sporadic sub-networks in the PM (Fig. 5b, Fig. S11d). Further investigation of the node genes in the core circadian gene-associated network revealed that the rhythmic node genes tended to show a wider expression breadth and longer gene length than non-rhythmic node genes (Fig. 5c, Fig. S11e, f). Moreover, the common node genes among the core circadian clock gene-associated interaction maps showed higher degrees than those in the AM- or PM-specific connectivity maps (Fig. 5d, e). Non-rhythmically expressed genes involved in the network showed higher transcriptional abundance than those outside of the network (Fig. 5f). Core circadian clock gene-associated RNAPII chromatin connectivity maps suggest that they are co-localized within the same “transcriptional factory” at AM circadian window and are released from the RNAPII-associated spatial gene clusters in the evening for coordinated transcriptional regulation.

## Discussion

During the past decade, a marked innovation in chromosome organization analysis has occurred, as genomic approaches for mapping chromatin interactions are yielding genome-wide chromatin interaction maps at unprecedented resolution. The circadian clock provides an ideal system for studying the temporal–spatial correlations among 3D genome organization, transcription, and physiology dynamics, representing the so-called 4D nucleome network [40]. In mammals, the plasticity in genome-wide chromatin folding during the circadian cycle and its role in oscillating transcription have been revealed [33, 36]. However, information on highly dynamic long-range chromatin interactions and their roles on transcriptional rhythmicity is lacking in plants. In this study, we mapped genome-wide chromatin interactions by using long-read ChIA-PET in rice and uncovered an RNAPII-mediated 3D genome architecture with marked changes at the genomic level of chromatin interactions, spatial clusters, and chromatin connectivity maps. Our findings suggest that widespread RNAPII-associated chromatin configuration is altered in a circadian manner and that these variations link chromatin architecture to coordinated rhythmic outputs. In mouse liver, Hi-C data showed topologically associating domains (TADs) harboring circadian genes that switch assignments between the transcriptionally active and inactive modules at different hours of the day; moreover, sub-TADs enriched in circadian genes exhibited extensive remodeling of enhancer-promoter interactions corresponding to their transcriptional activities, while their boundaries stably maintain their structure over time [33, 36]. ChIA-PET is known to be mediated by specific protein factors that tether linear genomic elements to higher-order chromatin structure, whereas Hi-C allows for probing of physical proximity between potentially any pair of genomic loci; thus, our ChIA-PET data provide functional specificity and a higher resolution for identifying any RNAPII-associated specific regulatory elements involved in interactions. We found that RNAPII-mediated chromatin interactions were abundant at the peak of *OsLHY* expression and then decreased at the opposite phase when *OsLHY* expression was low, in line with the previous findings that circadian gene promoters display a maximal number of chromatin contacts during their peak transcriptional output [31]. Furthermore, rhythmically expressed genes within the same peak phases of expression were preferentially tethered by RNAPII for coordinated transcription. Similar results were obtained using the mouse liver promoter-capture Hi-C dataset, where circadian genes, as well as contacted and transcribed regulatory elements, were found to reach maximal expression at the same time points [36]. Taken together, these findings suggest that the mechanism of chromatin conformation impacting rhythmic gene expression is conserved, at least in part, among mammals and plants.

Our comprehensive analysis also showed that RNAPII-interacting rhythmically expressed anchor genes were almost ubiquitously expressed genes, whereas tissue-specific genes were less likely to be rhythmically expressed; this is consistent with the observations drawn from the diurnal transcriptome atlas of a primate across tissues [41]. These results also support the notion that a primordial circadian system, as found in unicellular organisms, tunes the rhythmic expression of the majority of the genome to orchestrate the daily rhythms of essential biochemical and cellular processes. With the evolution of metazoans and tissue specialization, the basic set of genes necessary for cellular function was rhythmically expressed in all tissues and the circadian clock continued to oscillate in a similar phase [41]; however, non-rhythmically expressed genes were recruited for tissue-specific expression. During eukaryote evolution, a higher-order chromosomal organization emerged in the nuclei, providing a regulatory layer underlying circadian transcription. This would imply that the higher-order chromosomal organization for rhythmic transcription regulation is an ancient regulatory mechanism from the last eukaryote common ancestor.

In this study, we demonstrated that RNAPII (detected by antibodies with hypophosphorylated C-terminal domain) is recruited into the pre-initiation complex in a rhythmic manner, which is consistent with previous results showing that RNAPII recruitment is highly circadian in the mouse liver [11, 42]. It is worth noting that RNAPII-Ser5P occupancy is also circadian in mammals, indicating that both recruitment and initiation of RNAPII are under circadian control [11]. However, whether RNAPII-Ser5P, as well as RNAPII-Ser2P occupancy, is rhythmic in rice is unclear and remains to be verified. Given that rhythmic RNAPII recruitment preceded mRNA accumulation by approximately 2 h, other transcriptional and post-transcriptional mechanisms, such as circadian TF binding, chromatin remodeling, and post-transcriptional regulatory events may also contribute significantly to the generation of rhythmic mRNA in rice. It will be of interest to explore the role of circadian TFs and chromatin states in circadian regulation, where TF binding and histone deposition coupled with RNAPII occupancy to determine the ultimate genome-wide transcriptional outputs.

Overall, the results of this study illustrate the complexity and dynamics of chromatin structures during the circadian cycle; however, we only explored the RNAPII-mediated chromatin interacting complexes. Future studies will be needed to uncover the hierarchical organization of all clock regulators to determine the circadian chromatin architecture and decipher the molecular mechanisms implicated in the organization of the circadian interactome. Finally, we expect that the recapitulation of a structure-based framework with a greater diversity of time intervals and integrative analyses will be valuable in elucidating the mechanisms driving circadian genome organization, which will help determine the extent to which such remodeling contributes to circadian transcriptional regulation.

## Conclusions

In the present study, we mapped the 4D genome architecture of rice by employing improved ChIA-PET, a robust high-resolution 3D genome-mapping technology. Our results show that genome-wide rhythmic RNAPII occupancy is dynamically associated with diurnal RNAPII-mediated 3D genome architecture with marked changes at the genomic levels of chromatin interactions, spatial clusters, and chromatin connectivity maps throughout the day, and these variations link chromatin architecture to coordinate rhythmic outputs.

## Methods

### Plant materials and growth conditions

Rice *Xian*/*indica* cv. Minghui 63 (MH63) seeds were sown on May 19, 2017, in a paddy field. At 20 days after germination, the seedlings were transplanted into a paddy field of Huazhong Agricultural University at Wuhan, China, and grown under normal agricultural conditions. In the paddy field, the plants were transplanted at a density of one plant per 15 × 30 cm area. From July 7 to July 9, 2017 (natural long day conditions), 12 independent samples were collected (six time points at 4-h intervals per day for two successive days) and categorized into the following three groups: (1) samples for RNA isolation, which were immediately frozen in liquid nitrogen and stored at − 80 °C until use; (2) samples for chromatin immunoprecipitation followed by sequencing (ChIP-seq) and formaldehyde-assisted isolation of regulatory elements followed by sequencing (FAIRE-seq), which were cross-linked with 1% formaldehyde and stored at − 80 °C; and (3) samples for long-read chromatin interaction analysis by paired-end tag sequencing (ChIA-PET), which were double cross-linked with 1% formaldehyde and ethylene glycol bis [succinimidyl succinate] (EGS), and stored at − 80 °C. To avoid the effect of sampling (wounding response, etc.), individual plants were used only once for sampling.

### Whole transcriptome sequencing (RNA-seq) libraries preparation

RNA was isolated from the leaves using the RNeasy Plant Mini Kit (QIAGEN, 74904) according to the manufacturer’s instructions. 1.5 μg of total RNA was depleted of ribosomal RNAs using TruSeq Stranded Total RNA with Ribo-Zero Plant (Illumina, RS-122-2401) for RNA-seq according to the manufacturer’s instructions. RNA sequencing was performed on an Illumina HiSeq X Ten system (paired-end 150 bp reads).

### ChIP-seq libraries preparation

ChIP-seq was performed as previously described [43], with minor modifications. Rice mature leaves were cross-linked with 1% formaldehyde for 20 min and quenched with 0.2 M glycine at room temperature. Approximately, 0.5-g samples were used for each ChIP-Seq assay. Samples were ground into fine powder in liquid nitrogen and then lysed in 750 μl of Buffer S for 10 min at 4 °C. Then, the homogenate was added to 2.25 ml of Buffer F, mixed, and the chromatin was fragmented into 200–600 bp by sonication using a Bioruptor (Diagenode). Lysates were centrifuged at 20,000*g* for 10 min at 4 °C, and the supernatant was transferred to a new tube for ChIP. For the subsequent analysis, 20 μl of supernatant was used as an input sample. ChIP was performed using RNAPII antibody [43, 44] (BioLegend, 920102, unphosphorylated state of its large subunit C-terminal domain), which primarily recognized the pre-initiation complexes of RNAPII. First, 50 μl of Dynabeads® protein G beads (Life Technologies, 10003D) was washed with 300 μl of PBST buffer twice then resuspended in 200 μl of PBST buffer. Antibody (10 μl) was added to the beads and incubated for 6 h at 4 °C on a rotator. The antibody-bead complexes were washed with PBST twice and incubated with chromatin supernatant for 8 h or overnight on a rotator at 4 °C to immunoprecipitate the target chromatin. The immunoprecipitated chromatin was washed orderly with low-salt ChIP buffer, high-salt ChIP buffer, ChIP wash buffer, and TE buffer. One hundred microliters of freshly prepared ChIP Elution buffer was added to elute the protein-DNA complexes from beads, with agitation at 900 rpm for15 min at 65 °C. The eluate was then transferred to a new tube. Then, 5 μl of proteinase K was added to the eluate and incubated for 6 h at 55 °C to reverse-cross-linking the protein–DNA complexes. ChIP DNA was extracted by using phenol:chloroform:isoamyl alcohol (Sigma–Aldrich, P3803), precipitated with pre-cooled ethanol, and resuspended in TE buffer. ChIP DNA libraries were prepared using a NEBNext® Ultra™ II DNA Library Prep Kit for Illumina® (New England BioLabs, E7645). Finally, the DNA fragments were sequenced using an Illumina HiSeq X Ten system (paired-end 150 bp reads).

### FAIRE-seq libraries preparation

Nucleosome-depleted region mapping was performed using the FAIRE-seq method, with minor adaptations for rice [45, 46]. Approximately 0.5 g of mature rice leaves with cross-linked was used for each assay. Nuclei/chromatin isolation was performed as mentioned above. The final pellet was resuspended in 400 μl of sonic-FAIRE buffer (10 mM Tris-HCl, pH 8.0, 100 mM NaCl, 1 mM EDTA, 0.5% SDS, and protease inhibitor cocktail) and sheared to DNA fragments of 200–400 bp length by sonication in a Bioruptor (Diagenode). Lysates were centrifuged at 20,000*g* for 10 min at 4 °C, and the supernatant was transferred to a new tube. To prepare DNA that can be directly used for the identification of nucleosome-depleted regions, 1 volume of phenol:chloroform:isoamyl alcohol was added to the supernatant, vortex-mixed, and centrifuged at 20,000*g* for 10 min, and the supernatant was transferred to a new tube. These steps were repeated at least twice to remove nucleosome-coated DNA. After pre-cooled ethanol precipitation, DNA was resuspended in TE buffer. Subsequently, generated DNA fragments of 200–400 bp were generated using AMPure XP beads. FAIRE DNA library preparation and sequencing were performed as described above for ChIP-Seq.

### Long-read ChIA-PET library preparation

RNAPII-mediated ChIA-PET libraries were constructed according to the long-read ChIA-PET protocol with slight modifications [28, 44, 47]. In brief, mature rice leaves were cross-linked with 1% formaldehyde for 20 min. Next, EGS was added to a final concentration of 1.5 mM in PBS buffer and incubated for another 40 min at room temperature, after which glycine was added to a final concentration of 0.2 M to stop the cross-linking reaction. The samples were rinsed with ddH_2_O three times and stored at − 80 °C until further use. The sample (5 g) was ground into a fine powder in liquid nitrogen and resuspended in 100 ml of EB1 buffer. The homogenate was then filtered through Miracloth, and the filtrate was centrifuged at 1,800×*g* for 10 min at 4 °C. The pellet was washed three times in 5 ml of EB2 buffer and centrifuged at 2000×*g* for 10 min at 4 °C. Next, the pellet was washed in 2 ml of EB3 buffer and centrifuged at 2000×*g* for 1 h at 4 °C. The final pellet was resuspended in 1 ml of NLB buffer, and the chromatin solution was sonicated to achieve an average DNA size of 1–3 kb. After centrifugation at 2000×*g* for 10 min at 4 °C, the supernatant was transferred to a new tube for ChIP. The RNAPII antibody (80 μg; BioLegend; 920102) was mixed with 800 μl of suspended protein G magnetic beads and incubated at 4 °C for 8 h with rotation to coat the magnetic beads. All chromatin was extracted with antibody-loaded beads in a new tube and incubated at 4 °C overnight with rotation. The wash and library preparation steps were performed according to our previously published step-by-step long-read ChIA-PET library preparation protocol. The resulting library DNA products were then subjected to size selection and paired-end sequencing (2 × 150 bp) using the Illumina HiSeq X Ten system.

### RNA-seq data processing

The quality of raw data was controlled using FastQC, and the adapter sequences and low-quality reads were filtered out using Trimmomatic [48]. These strand-specific clean reads were mapped to the reference genome of MH63 using TopHat2 [49]. The expression level of each gene was quantified using HTSeq [50]. Normalization was performed using DESeq [51] according to the sequencing depth and then the formula *Ei*/*M* − 1 (*Ei* is the normalized counts of each time point, and *M* is the mean values of all time points) for row normalization. The final normalized values were used for the detection of rhythmic genes. A gene was defined as being expressed if the sum of count values of all time points was higher than a given value. The Cufflinks software was used to calculate the fragments per kilobase per million (FPKM).

### ChIP-seq data processing

After clipping adapters and trimming low-quality reads with Trimmomatic, the clean sequenced reads were aligned to the rice reference genome of MH63 using Burrows–Wheeler Aligner (BWA) with default parameters [52]. Samtools was used to remove potential PCR duplicates [53]. The reads with mapping quality > 30 were maintained. Only one uniquely aligning read per genomic position was retained for further analysis. RNAPII-occupied peaks were called using MACS2 [54] for each sample using input sample as a control. MACS2 was run with the default settings for RNAPII data calling the narrow-peak. For each identified protein-binding site, MACS2 uses the control data to calculate the *p* value, which is adjusted for multiple testing by a Benjamini–Hochberg correction. To further control the quality of each ChIP-seq dataset, the RSC was set to > 0.8 and the NSC was set to > 1.05, and FRiP was used to assess the peak quality [55]. To construct a union peak list from the six time points, bam files of two biological replicates after aligning were merged together using the command merge in samtools for peak calling, and these peaks from all time points were merged using bedtools mergeBed [56], and the regions of merged peaks were counted and normalized using the same method as RNA-seq processing. The final normalized values were used to predict rhythmic peaks.

### FAIRE-seq data processing

The process of FAIRE-seq data was similar to that of ChIP-seq data, except for the peak-calling settings using MACS2. For the FAIRE-seq data at each time point, MACS2 was run with “--nomodel --shift -100 --extsize 200” parameters.

### ChIA-PET data processing

The raw ChIA-PET data were processed using the modified ChIA-PET protocol [57], which is a software package for the automatic processing of ChIA-PET sequence data, including linker filtering, mapping tags to reference genomes, and identifying protein-binding sites and chromatin interactions. First, the linker filtering was carried out for the PET sequence reads. Next, the PET sequences were mapped to the MH63 rice reference genome using BWA with up to two mismatches allowed [52]. The mapped PETs were then used for further classification as self-ligation, inter-ligation, and other PETs. Inter-ligation PETs are those containing inter-chromosomal PETs defined as the head and tail of the PETs mapped onto different chromosomes, and intrachromosomal PETs are defined as the head and tail of the PETs mapped onto the same chromosome with a genomic distance of > 8 kb. In addition, we used the respective ChIP-seq peaks ± 10% regions as the given anchors to call clusters and identify specific chromatin interactions. To obtain more reliable results, only interactions with an FDR < 0.05 and PET count ≥ 5, determined by the sequencing depth, were considered for further analyses.

### Time-series data analysis for circadian cycling

After removing low signal values, normalized values across all time points of the RNA-seq, ChIP-seq, and FAIRE-seq data were analyzed to obtain the cycling time series using two programs: Bio_Cycle, which is based on deep-learning methods [37], and MetaCycle, which contains three algorithms, LS, ARSER, and JTK [58]. For Bio_Cycle, the period was set between 20 and 28 h [37]. Two independent days of sample collection were treated as biological replicates when statistically analyzing according to a guideline paper suggestion [59]. This experimental design can be advantageous when the focus of the study is rhythmicity under natural conditions, rather than isolated outputs of the circadian clock [59]. The parameters that describe the oscillations of signals and AMP from Bio_Cycle were used for further analysis, and the phase was estimated in MetaCycle. A rhythmic gene or peak was considered when the Bio_Cycle *q* value was < 0.05 after Benjamini–Hochberg correction, which is a commonly applied statistical threshold.

### Relationship between rhythmic RNAPII occupancy and gene expression

To analyze the correlation of rhythmic RNAPII occupancy and gene expression across all time points, we calculated the PCC between the rhythmic RNAPII peak signal and the corresponding rhythmic gene expression level. The average gene phase relative to the RNAPII occupancy phase was computed using the datasets which the phases of rhythmically expressed genes lag the phases of corresponding rhythmic RNAPII occupancy.

### Analysis of coexpression and phase difference of gene pairs

A gene was modified by RNAPII if the peak summit coordinates were within the gene body and + 500 bp regions. If a peak summit was in the region of two or more genes, we selected the gene with the highest FPKM value. The unique RNAPII-mediated interacting gene pairs were determined based on peak interactions.

The PCC was calculated for each gene pair based on the FPKM values across all time points, and the phase difference was calculated for each rhythmic gene pair. As controls, we randomly selected the same number of gene pairs with a similar distance distribution from all anchor and basal genes.

We downloaded the RNA-seq data of 20 rice tissues from GEO datasets and mapped the raw data to the reference genome of MH63 using hisat2 with “--dta-cufflinks” parameters [60]. The FPKM values were calculated using the Cufflinks software. Genes with FPKM > 1 were regarded as expressed genes. The number of tissues in which a gene was expressed was used to define the expression breadth.

### Analysis of chromatin spatial clusters (CSCs)

CSCs at 08:00 (AM) and 20:00 (PM) were classified into three types (static, AM-specific, and PM-specific) based on the degree ratio of AM and PM node genes. CSCs containing overlapping node genes with an AM:PM ratio > 3 and AM-specific node genes were defined as AM-specific CSCs, whereas CSCs containing overlapping node genes with a PM:AM ratio > 3 and PM-specific node genes were defined as PM-specific CSCs. The remaining CSCs with node genes with a degree fold-change ≤ 3 were defined as static CSCs. The PCC and the phase difference were calculated for all gene pairs in each CSC, and we randomly selected the same number of rewired gene pairs from anchor genes in the AM and PM as a control.

We computed the mean gene number of genes whose phase was around 08:00 and 20:00 with a high amplitude for each hub and randomly selected the same gene number from rhythmic and non-rhythmic sets as controls. All random procedures were repeated 1000 times.

### Construction of contact maps

We obtained the contact matrix using the bedpe2Matrix program of the ChIA-PET2 software [61] at 100 kb, 50 kb, and 10 kb resolutions with “--all --matrix-format complete” parameters from the ChIA-PET unique mapping reads, and the matrix was normalized by iterative correction and eigenvector decomposition using HiC-Pro [62].

### Motif mining

The FAIRE-seq peaks were merged across the six time points to construct a master peak list. Genes associated with time-specific chromatin loop were divided into different clusters (RR, RG–RG; RN/NR, RG–NG, or NG–RG; NN, NG–NG). To identify the phase-specific motif enriched in each cluster, the FAIRE-seq-merged peaks that overlapped with the promoters of rhythmic genes within a 2 kb window (+ 1 kb to − 1 kb relative to the annotated TSS) from each gene cluster were used for motif enrichment analysis using HOMER [63]. The remaining merged peaks that overlapped with non-rhythmic genes were used as the background. Only motifs that could be mapped to plants were included, and redundant motifs were removed.

### KEGG pathway analysis

The GATE-WAY (http://rice.hzau.edu.cn/rice/) was used for KEGG pathway enrichment analysis. The *p* value of a particular KEGG pathway item was calculated using Pearson’s chi-squared test and FDR correction, with an FDR cutoff of 0.05 as the significance threshold; the lower the *p* value, the more relevant the pathway.

### Correlation analysis of biological replicates

The rice genome was divided into 1-kb bins, and normalized values in each bin were calculated using deepTools software [64]. A scatter plot was generated based on the PCC between biological replicates, and a heatmap was plotted using the normalized values for each RNA-seq, ChIP-seq, and FAIRE-seq sample replicate.

### Data visualization

RNA-seq and ChIP-seq bigWig files were generated using the deeptools bamCoverage [64] function with the RPKM (reads per kilobase of transcript per million mapped reads) normalization; these files were then used for data visualization by IGV [65].

### Construction of RNAPII mediated gene networks

The interactome networks were constructed through one hop or two hops of all gene interactions originating from either seven or three core circadian genes at 08:00 and 20:00. For the connectivity networks of two hops, we merged the anchor genes of the interactome networks at the two points to produce a list of non-overlapping nodes. These were used to build the new networks. Nodes were connected on the basis of the interactions present in the ChIA-PET libraries respectively at the two points and visualized using Gephi [66]. Embedded meta-information was used for color coding.

### Statistical analysis

For comparison of multiple groups, statistical significance was calculated by the Kruskal–Wallis test. For comparison of two groups, we performed the Wilcoxon test; a *p* value of < 0.05 was considered statistically significant; NS indicates not statistically significant. Statistical parameters, including statistical analysis and statistical significance, are reported in the figure legends.

## Supplementary Information

**Additional file 1: Figure S1.** Summary of meteorological data in Wuhan from July 7, 08:00 to July 9, 08:00, 2017. **Figure S2.** Rhythmic occupancy of RNA polymerase II (RNAPII). **Figure S3.** Whole-transcriptome RNA-seq analysis of rhythmic gene expression in paddy field rice leaves. **Figure S4.** Reproducibility and loop span of RNAPII-mediated ChIA-PET data. **Figure S5.** Global patterns of chromatin interactions in rice. **Figure S6.** Characterization of RNAPII-mediated chromatin interactions. **Figure S7.** Percentage of overlap or dynamic anchors throughout the day and the correlation between peak intensity and transcription. **Figure S8.** Properties of RNAPII-organized chromatin spatial clusters (CSCs). **Figure S9.** Interaction frequencies around AM- and PM- specific node genes in the RNAPII-arranged networks. **Figure S10.** Features of node genes in the RNAPII-mediated networks. **Figure S11.** Core circadian clock genes-associated chromatin interaction networks.

**Additional file 2: Table S1.** Summary of RNAPII ChIP-seq libraries from the rice leaves.

**Additional file 3: Table S2.** Rhythmic RNAPII binding sites in rice leaves.

**Additional file 4: Table S3.** Summary of total RNA-seq libraries from the rice leaves.

**Additional file 5: Table S4.** Rhythmically expressed genes in rice leaves.

**Additional file 6: Table S5.** Summary of RNAPII ChIA-PET libraries from rice leaves.

**Additional file 7: Table S6.** RNAPII-mediated chromatin interactions at 08:00.

**Additional file 8: Table S7.** RNAPII-mediated chromatin interactions at 20:00.

**Additional file 9: Table S8.** Core circadian clock genes-associated chromatin interactions at 08:00 in the RNAPII-mediated chromatin connectivity maps.

**Additional file 10: Table S9.** RNA-Seq data of the different tissues used in this study.

**Additional file 11: Table S10.** Core circadian clock genes-associated chromatin interactions at 20:00 in the RNAPII-mediated chromatin connectivity maps.

**Additional file 12: Table S11.** Gene Expression Omnibus accession numbers.

**Additional file 13.** Review history.

## Data Availability

All the sequencing data generated in this study, including rhythmic RNA-seq, FAIRE-seq, RNAPII ChIP-seq, and RNAPII-mediated ChIA-PET data are deposited in the NCBI Gene Expression Omnibus (GEO) under the accession number GSE143724 [67]. Individual accession numbers are listed in Additional file 12: Table S11. All accessions of published RNA-seq data used in this study are provided in Additional file 10: Table S9.
