# Supplementary material for: Diurnal RNAPII-tethered chromatin interactions are associated with rhythmic gene expression in rice
Source: Genome Biol. 2022 Jan 6;23:7. doi: 10.1186/s13059-021-02594-7 (PMC8734370; doi:10.1186/s13059-021-02594-7)
Supplement: Supplementary file 1 — Additional file 1: Figure S1. Summary of meteorological data in Wuhan from July 7, 08:00 to July 9, 08:00, 2017. Figure S2. Rhythmic occupancy of RNA polymerase II (RNAPII). Figure S3. Whole-transcriptome RNA-seq analysis of rhythmic gene expression in paddy field rice leaves. Figure S4. Reproducibility and loop span of RNAPII-mediated ChIA-PET data. Figure S5. Global patterns of chromatin interactions in rice. Figure S6. Characterization of RNAPII-mediated chromatin interactions. Figure S7. Percentage of overlap or dynamic anchors throughout the day and the correlation between peak intensity and transcription. Figure S8. Properties of RNAPII-organized chromatin spatial clusters (CSCs). Figure S9. Interaction frequencies around AM- and PM- specific node genes in the RNAPII-arranged networks. Figure S10. Features of node genes in the RNAPII-mediated networks. Figure S11. Core circadian clock genes-associated chromatin interaction networks. [file 13059_2021_2594_MOESM1_ESM.docx]

**Diurnal RNAPII-tethered chromatin interactions are associated with rhythmic gene expression in rice**

**Supplemental Figures**

**Deng et al., 2021**


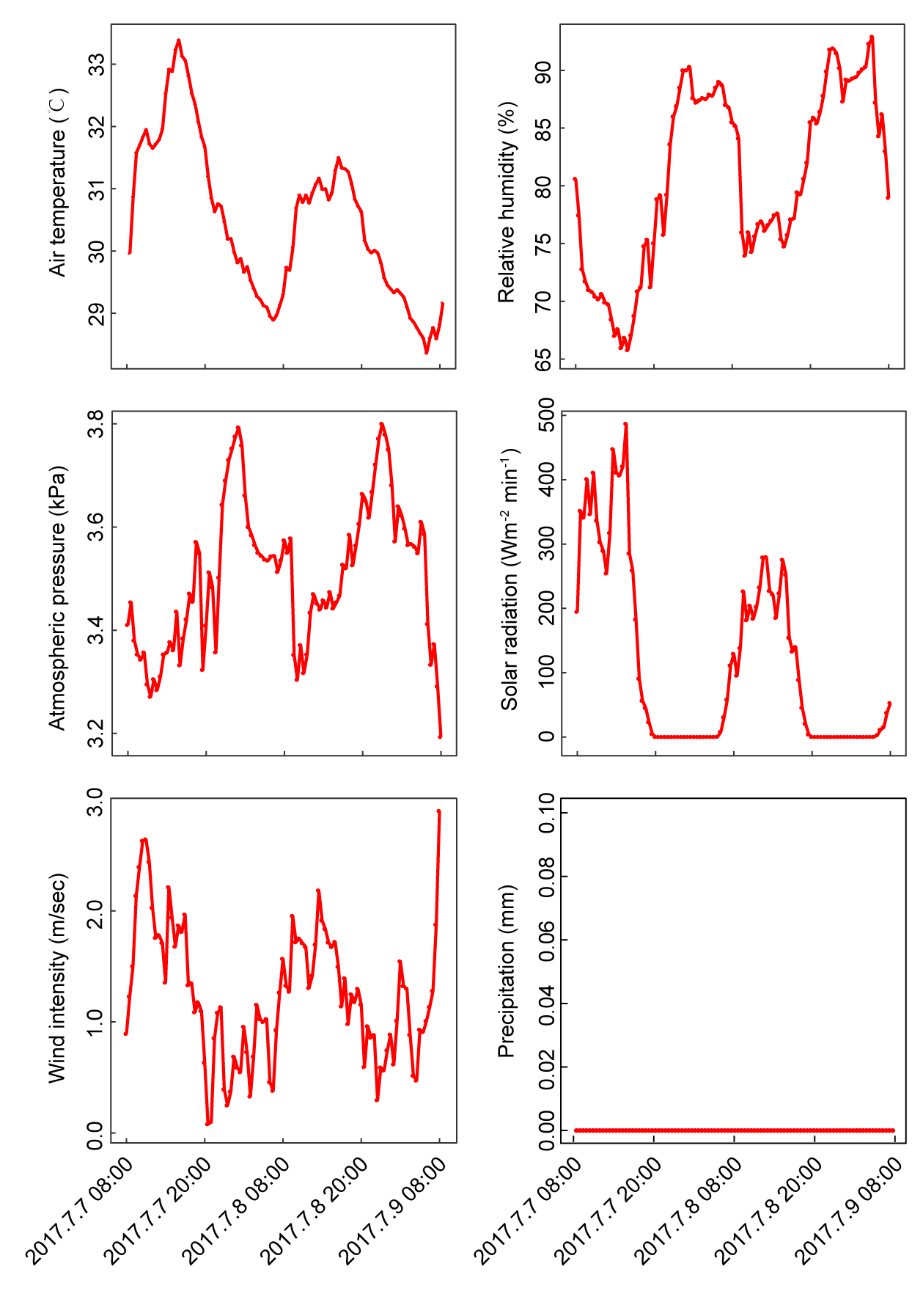


**Figure S1. Summary of meteorological data in Wuhan from July 7, 08:00 to July 9, 08:00, 2017.** Moving averages (window = 30 min) were plotted for air temperature (°C), relative humidity (%), atmospheric pressure (kPa), global solar radiation (Wm^-2^ min^-1^), and wind intensity (m/sec). Sums for each 30 min were plotted for precipitation (mm).


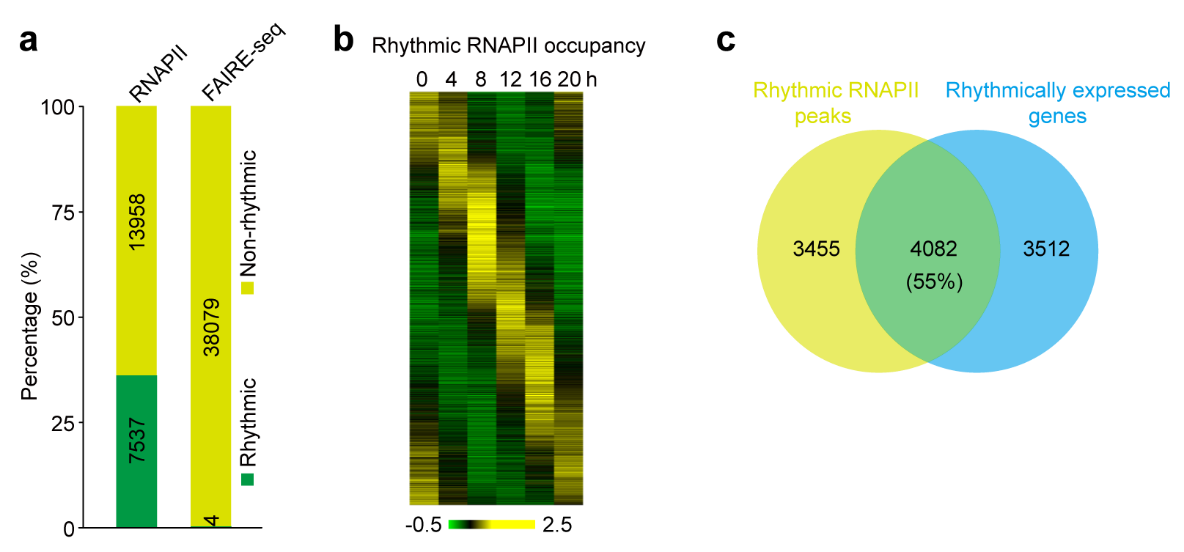


**Figure S2. Rhythmic occupancy of** **RNA polymerase II (RNAPII).**

(**a**) Distribution of RNAPII occupancy and formaldehyde-assisted isolation of regulatory elements followed by sequencing (FAIRE-seq) peak showing rhythmic (green) and non-rhythmic (yellow) patterns throughout the day.

(**b**) Heatmap of rhythmic RNAPII occupancy across six time points. Each RNAPII binding site is represented as a horizontal line, ordered vertically by phase determined by MetaCycle.

(**c**) Venn diagram showing the overlap of rhythmic RNAPII binding sites and rhythmically expressed genes.


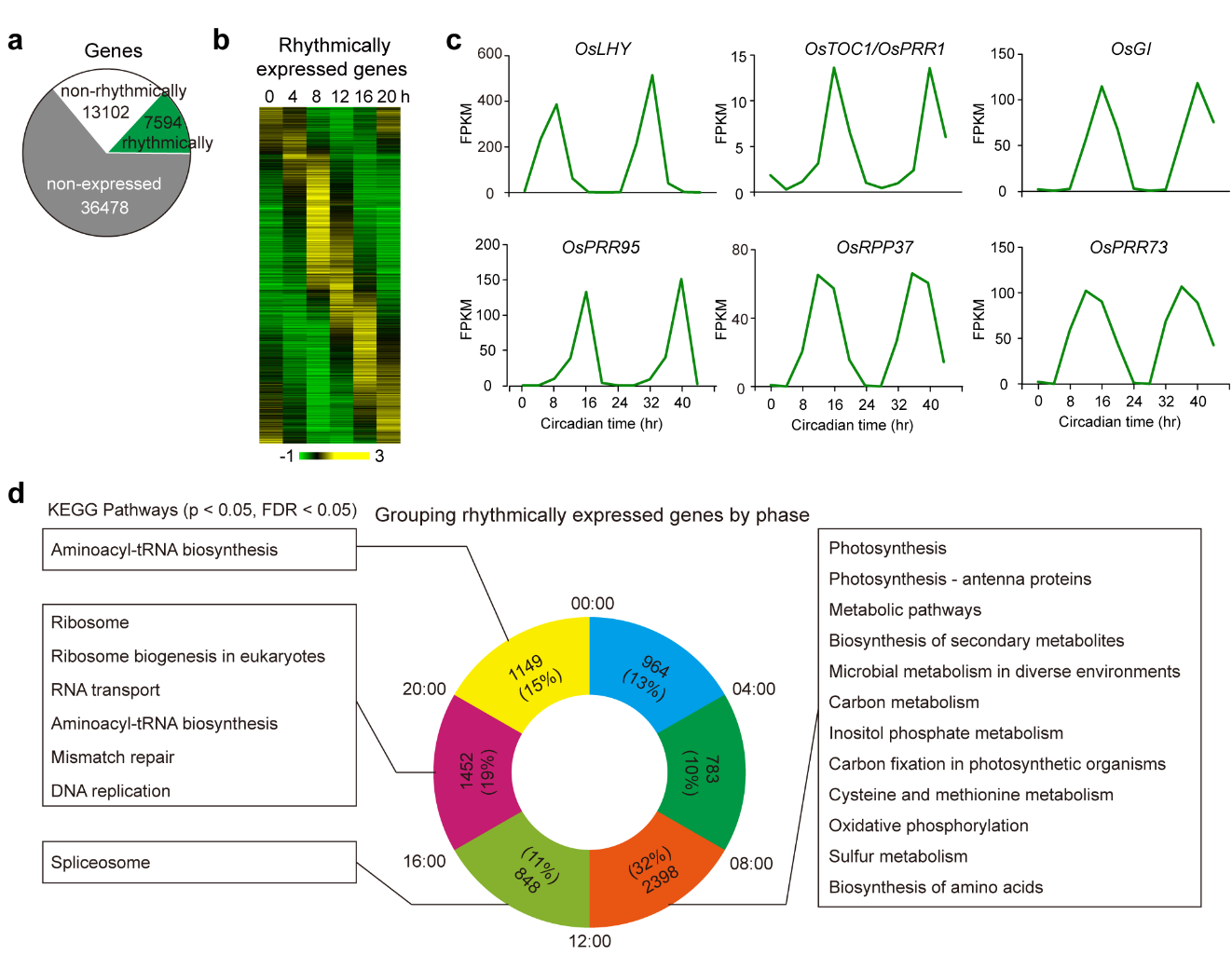


**Figure S3. Whole-transcriptome RNA-seq analysis of rhythmic gene expression in paddy field rice leaves.**

(**a**) Number of rhythmically, non-rhythmically, and non-expressed genes.

(**b**) Heatmap of rhythmically expressed genes across six time points. Each gene is represented as a horizontal line, ordered vertically by the phase determined by MetaCycle.

(**c**) RNA-seq read coverage in FPKM reads. The expression of *OsLHY*, *OsTOC1*/*OsPRR1*, *OsGI*, *OsPRR95*, *OsPRR37*, and *OsPRR73* show circadian patterns (Bio_Cycle; *q*-value < 0.05).

(**d**) Most significantly enriched KEGG pathways (FDR < 0.05) for the rhythmically expressed genes grouped by their phases; the six groups of rhythmically expressed genes are labeled in different colors.


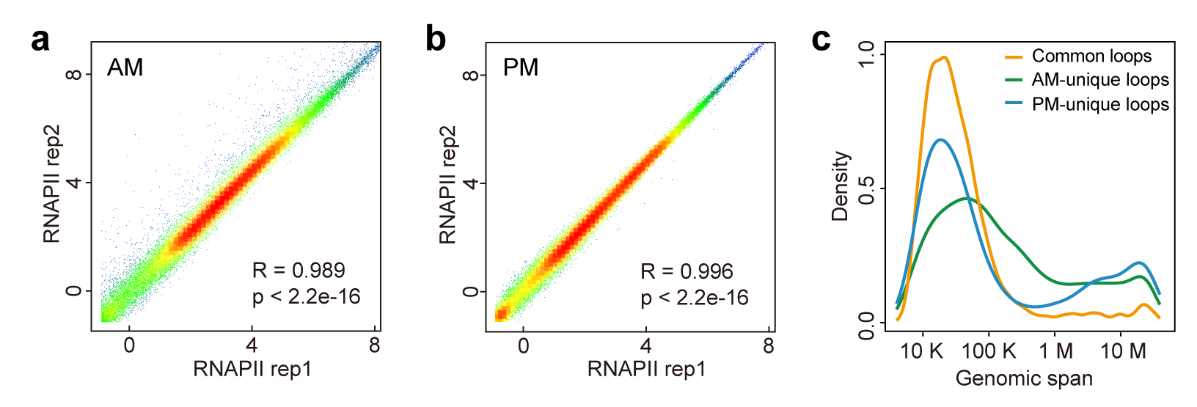


**Figure S4. Reproducibility and loop span of RNAPII-mediated ChIA-PET data.**

(**a**,**b**) Scatter plots showing the reproducibility of RNAPII-mediated ChIA-PET data at 08:00 (AM) (**a**) and 20:00 (PM) (**b**) between two replicates. Spearman correlation coefficients are shown.

(**c**) Loop span distribution of RNAPII-associated intrachromosomal interactions.


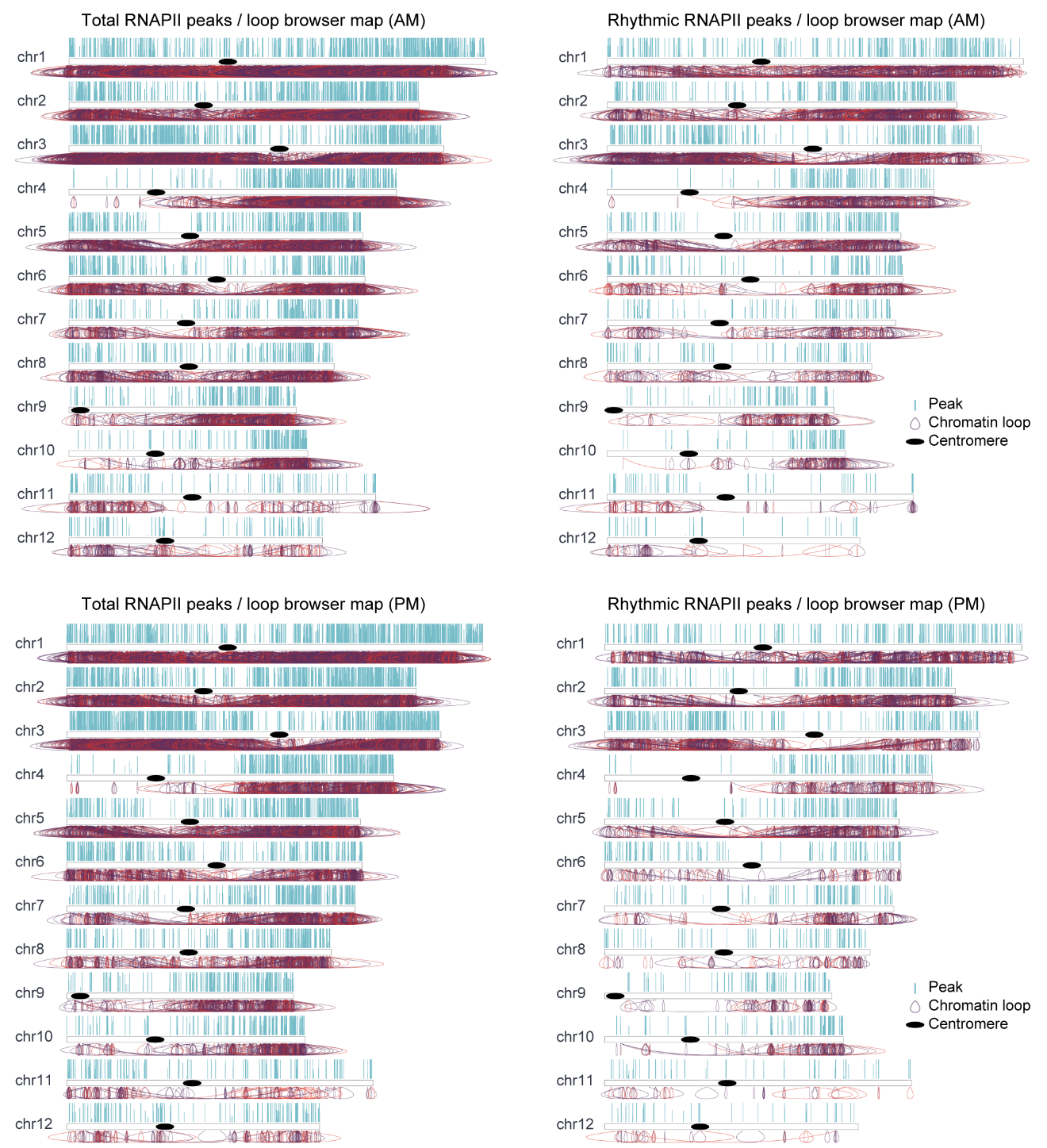


**Figure S5. Global patterns of chromatin interactions in rice.**

Global views of total (left) and rhythmic (right) RNAPII-associated intrachromosomal interactions at 08:00 (top) and 20:00 (bottom). Blue peaks above the chromosomes indicate binding sites, and red curves under chromosomes indicate interactions. A deeper red color indicates stronger interactions.


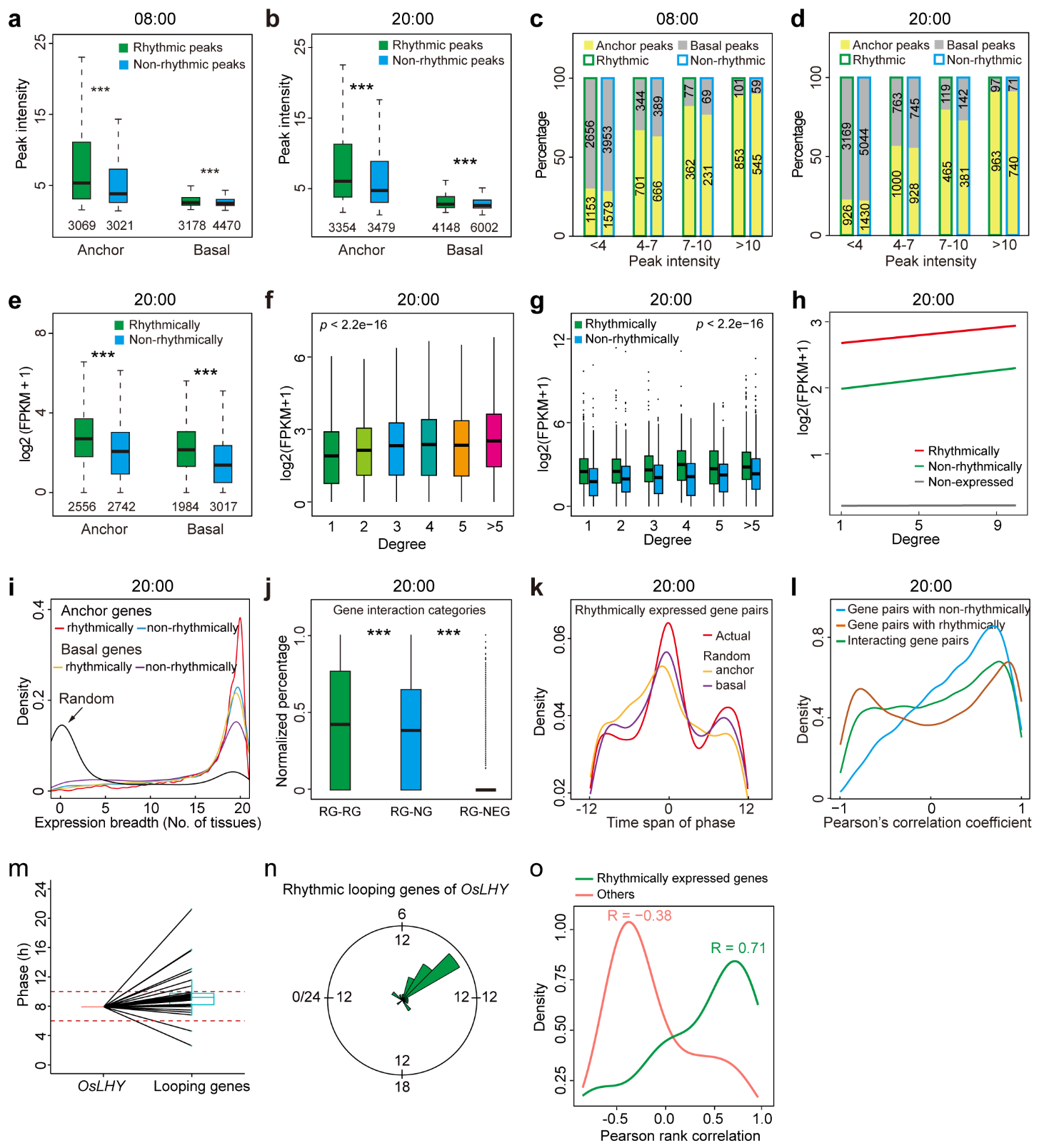


**Figure S6. Characterization of RNAPII-mediated chromatin interactions.**

(**a**, **b**) Boxplot showing the intensity of rhythmic and non-rhythmic RNAPII peaks involved or not involved in chromatin interactions at 08:00 (**a**) and 20:00 (**b**). ****p* < 0.0001.

(**c**, **d**) Rhythmic peaks with higher peak intensity tended to be anchor peaks at 08:00 (**c**) and 20:00 (**d**).

(**e**) Boxplot showing expression levels of rhythmically and non-rhythmically expressed genes involved or not involved in RNAPII-mediated chromatin interactions at 20:00. ****p* < 0.0001.

(**f**) Boxplot showing positive correlations between the degrees and expression levels of anchor genes at 20:00.

(**g**) Boxplot showing the positive relationship between degrees and transcriptional abundance of rhythmically and non-rhythmically expressed anchor genes at 20:00.

(**h**) With an increase in degree, rhythmically expressed anchor genes showed higher transcript abundance than did non-rhythmically expressed anchor genes did at 20:00. The correlation coefficient between transcript abundance and degree among rhythmically, non-rhythmically, and non-expressed genes was 0.55, 0.62, and 0.01, respectively. Rhythmically vs. non-rhythmically, *p*-value = 0.4069 (binomial test).

(**i**) Expression breadth (number of tissues in which a gene is expressed) of RNAPII-mediated anchor and basal genes with rhythmic or non-rhythmic characteristic at 20:00. Random genes served as controls.

(**j**) Distribution of rhythmically expressed gene-centric interactions at 20:00. The percentages of RG (rhythmically expressed genes)–RG, RG–NG (non-rhythmically expressed genes), and RG–NEG (non-expressed genes) interactions are listed. ****p* < 0.0001.

(**k**) Distribution of phase spans of RG–RG interaction gene pairs and randomly picked gene pairs from rhythmically expressed anchor genes and rhythmically expressed basal genes in the 20:00 datasets. Actual vs. random anchor, *p*-value = 0.001196; actual vs. random basal, *p*-value = 0.7287; random anchor vs. random basal, *p*-value = 0.1085 (Kolmogorov–Smirnov test).

(**l**) Distribution of Pearson’s correlation coefficient (PCC) values for RNAPII-bound interacting gene pairs, gene pairs with a loop connecting rhythmically expressed genes, and gene pairs with a loop connecting non-rhythmically expressed genes in the 20:00 datasets.

(**m**) Phase distribution of *OsLHY* and *OsLHY*-associated chromatin loop connecting genes in the RNAPII-mediated chromatin connectivity maps. The red dashed lines indicate the temporal window between 06:00 and 10:00.

(**n**) Phase distribution of *OsLHY*-associated chromatin loop connecting genes in the RNAPII-mediated chromatin connectivity maps represented as a rose plot. The values inside the circle represent the coordinates, the numbers outside the circle indicate the sidereal hours.

(**o**) Correlation analysis between *OsLHY* expression level and *OsLHY-*looped rhythmic genes or other genes (non-rhythmic and non-expressed genes).


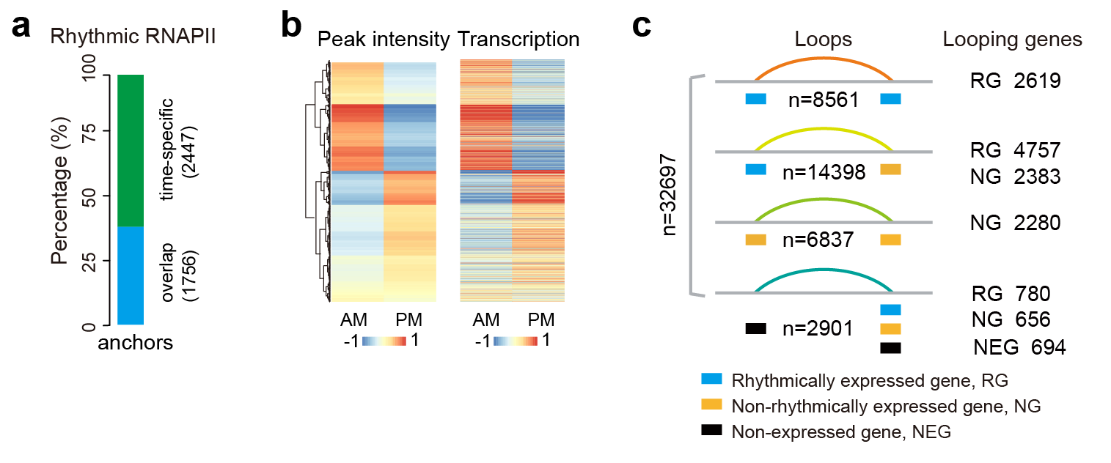


**Figure S7. Percentage of overlap or dynamic anchors throughout the day and the correlation between peak intensity and transcription.**

(**a**) Distribution of overlap (common) or time-specific rhythmic RNAPII anchor peaks throughout the day.

(**b**) Heat map representation of hierarchical clustering of time-specific rhythmic RNAPII peak intensity and levels of the corresponding transcripts. Each RNAPII occupancy and the corresponding gene is represented as a horizontal line.

(**c**) Characterization of RNAPII-mediated time-specific chromatin loops and looping genes.


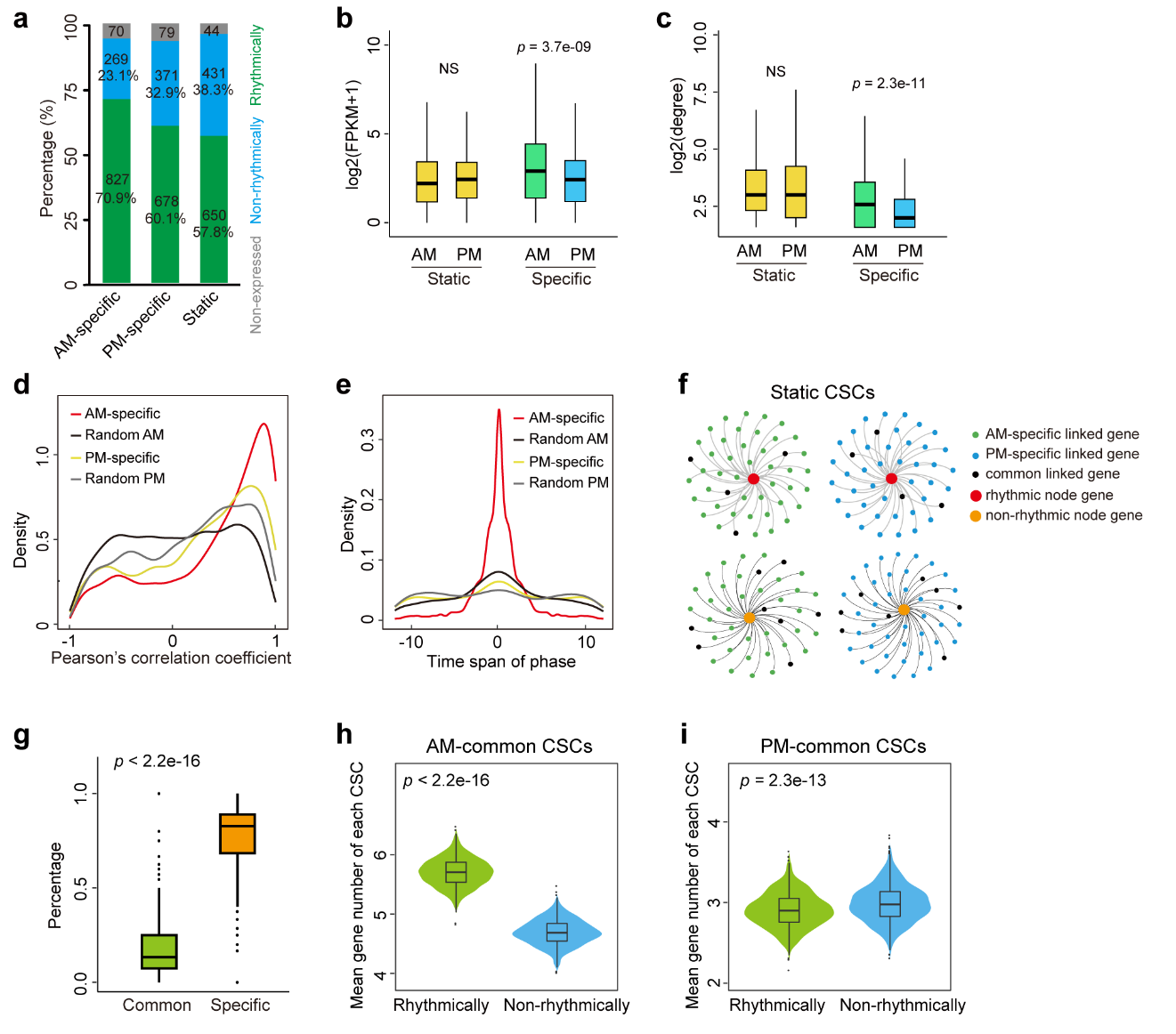


**Figure S8. Properties of RNAPII-organized chromatin spatial clusters (CSCs).**

(**a**) Distribution of rhythmically, non-rhythmically, and non-expressed node genes across the three types of CSCs: (i) AM-specific state, (ii) PM-specific state, and (iii) static state (overlap).

(**b**) Boxplot showing the expression levels of node genes involved in the AM-specific state, PM-specific state, and static state (overlap) CSCs. NS, no significant difference.

(**c**) Boxplot showing the degrees of node genes involved in the AM-specific state, PM-specific state, and static state CSCs. NS, no significant difference.

(**d**) Distribution of PCC values for AM-specific, PM-specific, and randomly selected gene pairs from control regions with the same genomic span and gene density distribution.

(**e**) Distribution of time spans between anchor gene peak expression phases for AM-specific, PM-specific, and randomly selected gene pairs.

(**f**) In the static CSCs, the rhythmically and non-rhythmically expressed node gene-centric interaction map changed throughout the day. The rhythmically expressed node gene MH01g0488600 was linked to 47 and 45 genes in the AM and PM datasets, respectively (top). The non-rhythmically expressed node gene MH07g0016400 was linked to 42 and 47 genes in the AM and PM datasets, respectively (bottom).

(**g**) Rhythmic properties of static node gene-connecting targets. Static node genes shared about 10% common linked genes and possessed 90% AM or PM-specific linked genes.

(**h**, **i**) Violin plot showing the distribution of mean gene number in AM-common (**h**) and PM-common (**i**) CSCs with randomly simulated rhythmically and non-rhythmically expressed genes.


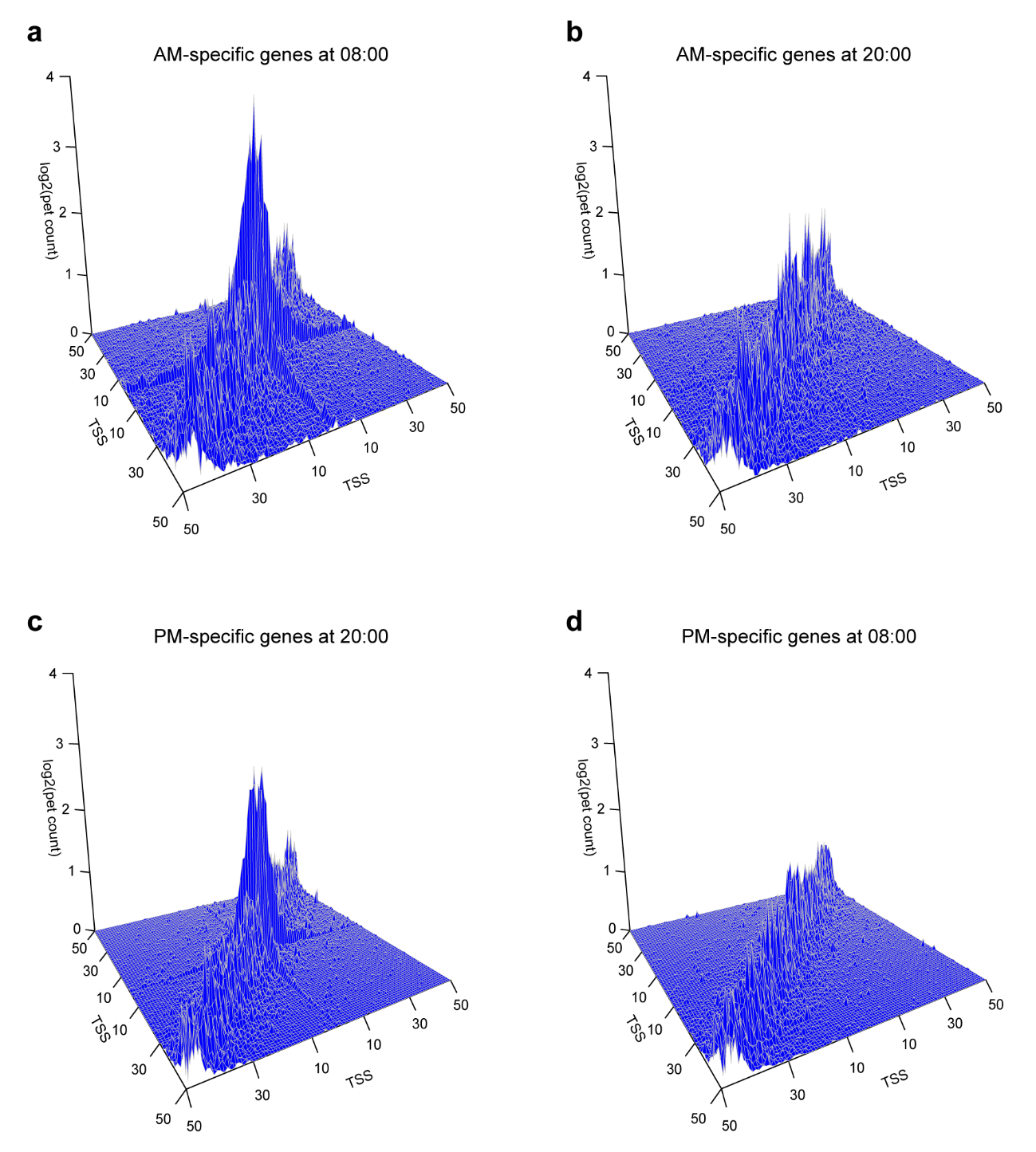


**Figure S9.** **Interaction frequencies around AM- and PM- specific node genes in the RNAPII-arranged networks.**

(**a**, **b**) Three-dimensional profiles of average interaction frequencies in 100 linear genes around AM-specific node genes in the 08:00 (**a**) and 20:00 (**b**) datasets. Profiles are centered at the transcriptional start site (TSS).

(**c**, **d**) Three-dimensional profiles of average interaction frequencies in 100 linear genes around PM-specific node genes in the 20:00 (**c**) and 08:00 (**d**) datasets. Profiles are centered at the TSS.


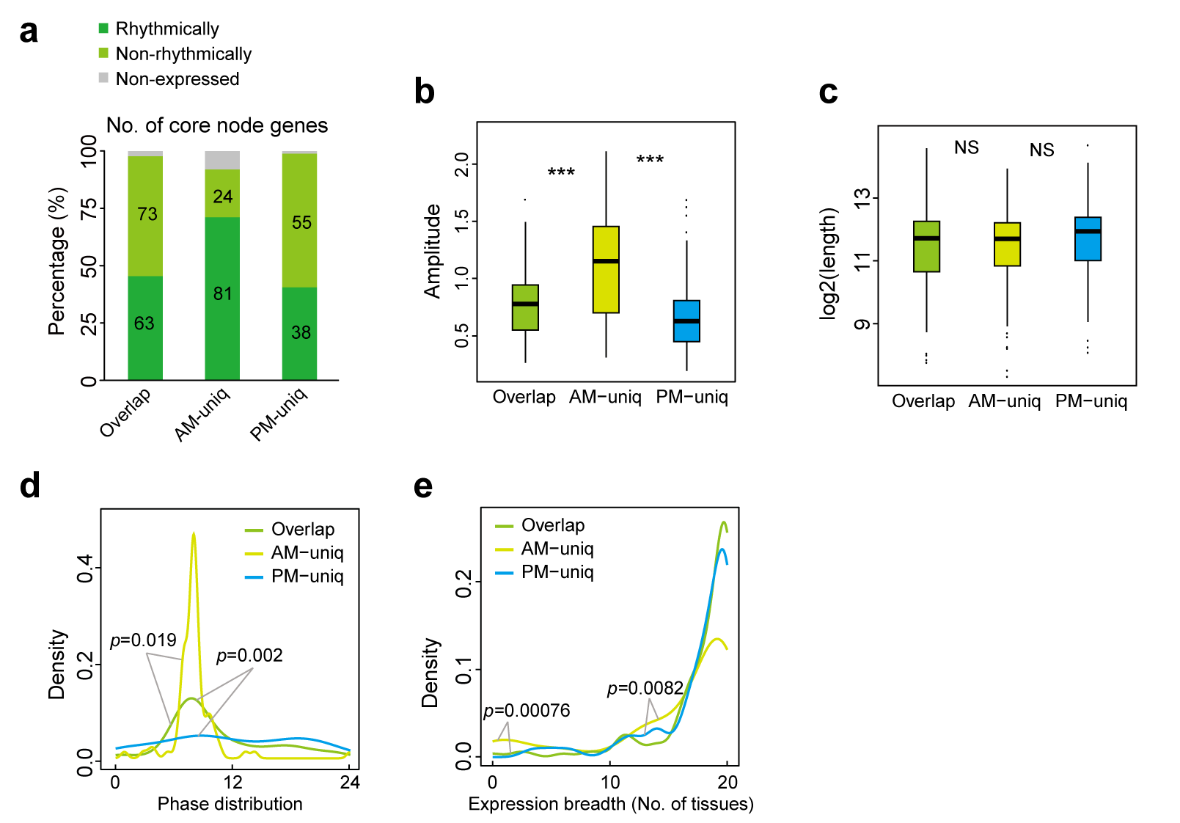


**Figure S10. Features of node genes in the RNAPII-mediated networks.**

(**a**) Distribution of three types of core node genes (degree ≥ 32): AM-unique, PM-unique, and overlap node genes.

(**b, c**) Boxplots showing the amplitudes (**b**) and gene lengths (**c**) of three types of core node genes. ****p* < 0.0001. NS, no significant difference.

(**d, e**) Peak phase distribution (**d**) and expression breadth (**e**) of three types of core node genes.


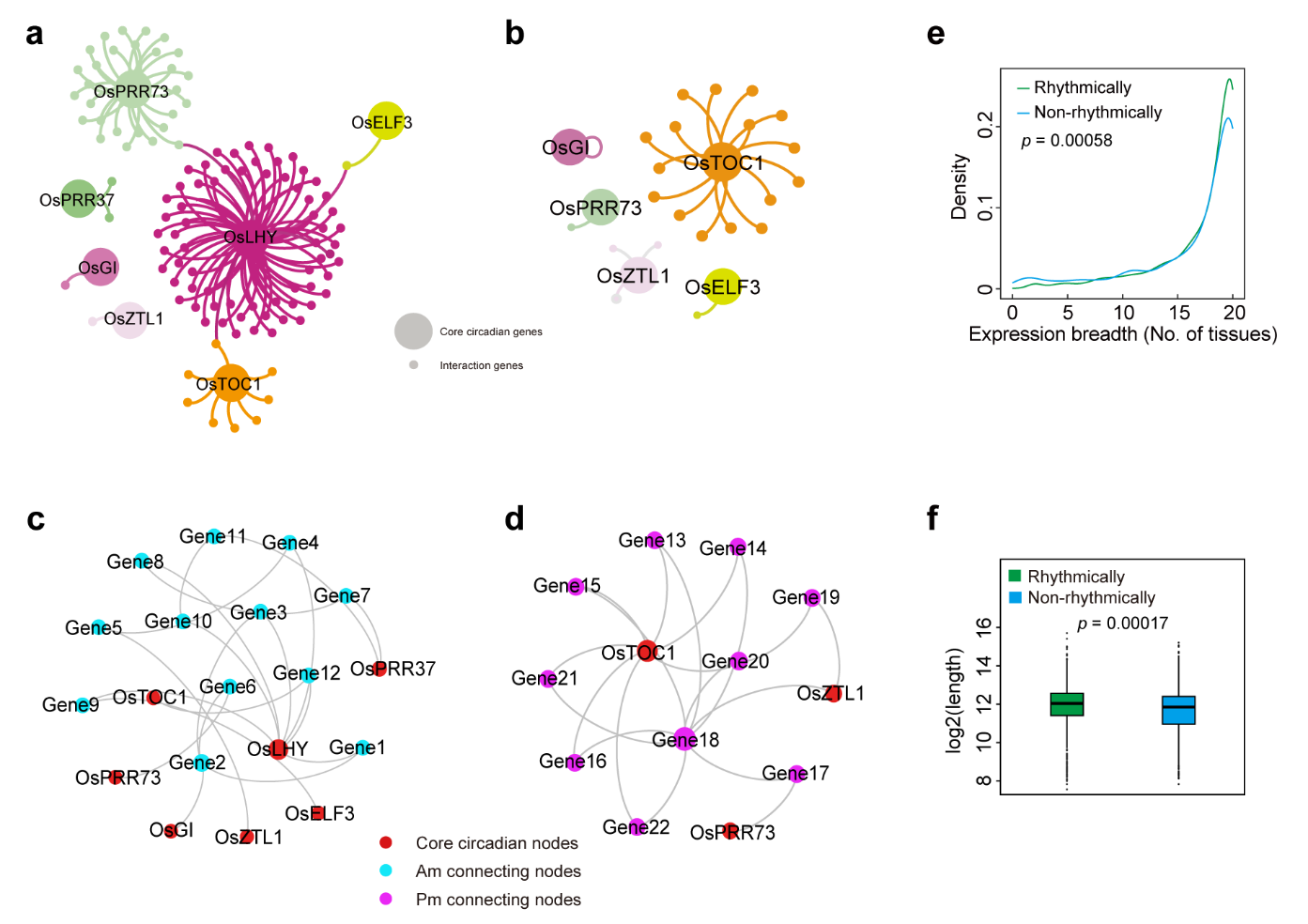


**Figure S11. Core circadian clock genes-associated chromatin interaction networks.**

(**a, b**) Connectivity was constructed of one-hop interactions mediated by core circadian genes at 08:00 (**a**) and 20:00 (**b**).

(**c, d**) Connectivity framework constructed of two-hop interactions connecting the core circadian clock genes at 08:00 (**c**) and 20:00 (**d**).

(**e**) Expression breadth of rhythmic and non-rhythmic node genes in the core circadian genes-associated network.

(**f**) Boxplots showing the gene length of rhythmic and non-rhythmic node genes in the core circadian genes-associated network.
