# Supplementary material for: Diurnal RNAPII-tethered chromatin interactions are associated with rhythmic gene expression in rice
Source: Genome Biol. 2022 Jan 6;23:7. doi: 10.1186/s13059-021-02594-7 (PMC8734370; doi:10.1186/s13059-021-02594-7)
Supplement: Supplementary file 13 — Additional file 13. Review history. [file 13059_2021_2594_MOESM13_ESM.docx]

Review History

**First round of review**

**Reviewer 1**

**Are you able to assess all statistics in the manuscript, including the appropriateness of statistical tests used?**

No, I do not feel adequately qualified to assess the statistics.

**Comments to author:**

Many genes show expression rhythm in plants, but people don't know whether chromatin interactions rhythmically oscillate in corresponding genes. In this study, Deng et al explored the rhythmic patterns of gene expression, RNAPII accupancy and chromatin interactions in rice. They observed high correlation between expression and RNAPII accupany and found RNAPII occupancy preceded mRNA accumulation by about 2 hours. The rhythmically expressed genes were enriched in RNAPII-associated CSCs in morning but non-rhythmically expressed genes were enriched in CSCs in evening. Overall, it is an interesting and comprehensive study.

Comments:

1 My biggest concern is that ChIA-PET in this study only captures RNAPII-associated chromatin interactions. The rhythm of chromatin interactions in some genes may be just due to enrichment of RNAPII in ChIA-PET. Not sure whether the whole chromatin interactions rhythmically oscillate in these genes. It may be better to explore rhythmic pattern of chromatin interactions using Hi-C.

2 Only 37% of active genes show expression rhythm, but why most chromatin loops (91%) were time-specific? Are most non-rhythmic genes not looped by RNAPII-associated chromatin interactions? It is a little strange. More discussion and explanation are needed.

3 Most rhythmically expressed genes are in morning phase (Fig. 1d). Is it common for plant? Is the gene number in morning phase much bigger than the gene number in evening phase in other species? Any explanation?

4 I don't understand why the dash lines don't locate in the peak of density in Fig. 1f. Is it an error?

5 The comma is used to separate groups of thousands in some numbers (line 133, 20,667), but not in whole manuscript (line 134, 4526). It is better to use a consistent format in whole manuscript.

6 Line 297-298, the statistic analysis should be used to check whether the difference is significant.

**Reviewer 2**

**Are you able to assess all statistics in the manuscript, including the appropriateness of statistical tests used?**

No, I do not feel adequately qualified to assess the statistics.

**Comments to author:**

This study investigated how RNAPII dynamically interacts with their corresponding regulatory regions during the circadian cycle. Long-read chromatin interaction analysis (ChIA-PET) was used to analyze genome-wide chromatin interactions associated with RNAPII in rice. The interplay between 3D genome architecture, rhythmic RNAPII occupancy, and rhythmic gene expression was also analyzed. The generated RNAPII-associated high resolution dynamic chromatin interactome maps provides new insight for understanding orchestrated circadian transcriptional outputs. The experiments in this study is carefully designed and well conducted. I have the following comments.

1. RNAPII signals increased sharply at transcription start sites (TSS), decreased on entering gene body regions, and peaked at polyadenylation site (PAS) (page5, line 106).

Please explain why RNAPII signals peaked at polyadenylation site.

2. In total, we identified 20,667 high-confidence (FDR < 0.05) RNAPII-associated chromatin interactions connecting 4526 genes at 08:00 (Fig. 3a) and 21,001 loops connecting 5298 genes at 20:00 (Additional file 1: Figure S6e) (Page 6, line 132).

Please indicate how much overlap between the RNAPII-associated chromatin interactions connecting 4526 genes at 08:00 and those 5298 genes at 20:00.

3. OsLHY, a well-known core circadian clock gene in rice [38], was found to form loops with 74 genomic regions at 08:00 in the RNAPII-mediated chromatin connectivity maps (Fig. 2e, f) (Page 6, line 142).

The authors may want to describe in more details about those74 genomic regions associated with OsLHY (eg., there are any specific loci that maybe functional associated with OsLHY). In addition to OsLHY, I would like to see the interacting profiles of other core circadian clock genes such as OsTOC1, OsPRR73, and OsELF3. Do they share similar interacting profiles?

4. In contrast, these RNAPII tethered chromatin loops were virtually undetectable at 20:00, which was the time point of trough OsLHY expression (Fig. 2e)About 34% (569/1631) of jmj705-upregulated and 27% (282/1055) of jmj705 downregulated genes overlapped with those detected in ossnrk1a1 under starvation (Fig 2C) (Page 6, line 145).

It appears that there are still some RNAPII tethered chromatin loops at 20:00. Please indicate clearly whether the RNAPII tethered chromatin loops at 20:00 are completely different from those at 8:00.

5. Among the 13,738 and 16,983 RNAPII-binding sites detected at 08:00 and 20:00, respectively, 4 6090 (44%) and 6883 (41%) served as chromatin interaction anchors, respectively (Page 7, line 153).

Please indicate how much overlap among the 13,738 and 16,983 RNAPII-binding sites detected at 08:00 and 20:00.

6. Rhythmically expressed node genes within static CSCs with expression peaks were relatively randomly distributed throughout the day and had similar expression levels in the morning and evening (Fig. 4c, d, Additional file 1: Figure S8a) (Page 9, line 217).

In Fig. 4c, it appears to me that the rhythmically expressed node genes within static CSCs showed higher levels of expression in the morning than in the evening. Please explain.

7. Further investigation of the core node genes in the core circadian gene-associated network revealed 297 that the rhythmic core node genes tended to show a wide expression breadth and short gene length (Fig. 5c) (Page 12, line 295).

Please explain what is the meaning of "a wide expression breadth and short gene length".

8. Moreover, the common node genes among the core circadian clock genes associated with the AM and PM interactome maps showed higher degrees than those in the AM- or PM-specific connectivity maps (Fig. 5d, e) (Page 13, line 298).

Please explain what is the meaning of "showed higher degrees".

9. The expression of TOC1 is repressed at morning. However, TOC1 appeared in both AM and PM specific groups in Fig 5. In addition, the evening genes ELF3 and GI both appeared in the AM specific group in Fig 5. These results need to be explained.

**Authors Response**

**Point-by-point responses to the reviewers’ comments:**

*We sincerely thank the reviewers and the editor for their valuable comments and kind considerations. We have revised our manuscript according to the comments which have significantly improved our manuscript. Please find point-by-point response to each comment below.*

Reviewer #1: Many genes show expression rhythm in plants, but people don't know whether chromatin interactions rhythmically oscillate in corresponding genes. In this study, Deng et al explored the rhythmic patterns of gene expression, RNAPII occupancy and chromatin interactions in rice. They observed high correlation between expression and RNAPII accupany and found RNAPII occupancy preceded mRNA accumulation by about 2 hours. The rhythmically expressed genes were enriched in RNAPII-associated CSCs in morning but non-rhythmically expressed genes were enriched in CSCs in evening. Overall, it is an interesting and comprehensive study.

Comments:

1 My biggest concern is that ChIA-PET in this study only captures RNAPII-associated chromatin interactions. The rhythm of chromatin interactions in some genes may be just due to enrichment of RNAPII in ChIA-PET. Not sure whether the whole chromatin interactions rhythmically oscillate in these genes. It may be better to explore rhythmic pattern of chromatin interactions using Hi-C.

*Response: We understand the reviewer’s concern. The key reason of using ChIA-PET is that this method could map rhythmic chromatin interactions at very high resolution (which are typically <100 bp at peak summit) and allows us to explore the mechanism of transcriptional regulation of DNA regulatory elements. However, rice Hi-C data published so far usually showed the contact heatmaps of TADs and compartments, both of which are higher-level topological architectures at low-resolution level, rather than the specific interactions between regulatory elements, such as gene-gene interactions. Thus, it is kind of a tradeoff between the comprehensiveness and resolution of interacting loci, although we may lose some of interactions, we still could capture most of the promoter–promoter interactions tethered by RNA polymerase II (RNAPII) that contribute to rhythmic gene expression in our study.*

*ChIA-PET produces unique and accurate data for detecting specific chromatin interactions mediated by protein factors in high resolution and presents more details for detecting the 3D genome organization and function. By adopting the long-range ChIA-PET methods, we have mapped H3K4me3- and RNAPII-associated promoter–promoter interactions at nucleotide/gene resolution in rice and maize (Zhao et al. 2019; Peng et al. 2019). In this study, RNAPII occupancy was rhythmic and rhythmic RNAPII peak intensity was significantly correlated with rhythmic transcript expression levels (Pearson rank correlation = 0.69, over the six-time points; Fig. 1e, Additional file 1: Figure S2c). Based on these findings, we used long-range ChIA-PET to investigate how RNAPII dynamically interacted with their corresponding regulatory regions and orchestrated circadian transcriptional outputs during the circadian cycle. Rhythmic RNAPII binding dynamically correlated with RNAPIImediated chromatin architecture remodeling as well as the rhythmic expression patterns of looping genes.*

2 Only 37% of active genes show expression rhythm, but why most chromatin loops (91%) were timespecific? Are most non-rhythmic genes not looped by RNAPII-associated chromatin interactions? It is a little strange. More discussion and explanation are needed.

*Response: We thank the reviewer for the suggestion. We defined the chromatin loops which connect same anchor gene pairs at 08:00 and 20:00 datasets as the common loops; chromatin loops which connect either one or both different anchor genes at 08:00 and 20:00 datasets as the time-specific loops. 32,697 time-specific chromatin loops mean that there are 32,697 chromatin loops that looping different genes in the morning and evening. Actually, for 32,697 time-specific chromatin loops, ~26% (n = 8,561) were RG (rhythmically expressed genes)–RG interactions and looped 2,619 rhythmically expressed genes; ~44% (n = 14,398) were RG–NG (non-rhythmically expressed genes) or NG–RG interactions, and looped 4,757 rhythmically expressed genes and 2,383 non-rhythmically expressed genes, respectively; ~21% (n = 6,837) were NG–NG interactions and looped 2,280 non-rhythmically expressed genes; ~9% (n = 2,901) were NEG (non-expressed genes)-associated interactions, and of all the looping genes, 37% (780), 31% (656), and 32% (694) were associated with rhythmically, non-rhythmically, and non-expressed genes, respectively (Additional file 1: Figure S7c). In total, there are 3,259 rhythmically (43%, out of 7,594 rhythmically expressed genes), 3,241 non-rhythmically (25%, out of 13,102 non-rhythmically expressed genes), and 694 non-expressed (2%, out of 36,478 non-expressed genes) genes, respectively, were involved in RNAPII-associated time-specific chromatin interactions. These results suggest that some of non-rhythmically expressed genes were also looped by RNAPII-associated time-specific chromatin interactions. We have added corresponding explanation in the revised manuscript (lines 197- 209, Additional file 1: Figure S7c).*

3 Most rhythmically expressed genes are in morning phase (Fig. 1d). Is it common for plant? Is the gene number in morning phase much bigger than the gene number in evening phase in other species? Any explanation?

*Response: The reviewer raised an interesting comment. We followed the reviewer’s idea and collected published rhythmic transcriptome data from different plant species, including Arabidopsis (Romanowski et al. 2020), barely (Muller et al. 2020), rapeseed (Greenham et al. 2020), and soybean (Li et al. 2019). Some of published data could not be accessible. According to experiments conditions, there are three types of transcriptome data: 1) the data from fluctuating field conditions termed as MH63_NLD (generated in this study), 2) from constant light (LL), plants under 12 hr light/12 hr dark before transfer to constant light, which is the conventional manipulation for studying intrinsic rhythmicity, the data named as Arabidopsis_LL, rapeseed_LL, and soybean_LL. 3) driven conditions (e.g., light:dark [LD]), the data named as MH63_LD (unpublished data in our lab) and Barely_LD. By using the same data analysis pipeline, we plot the phase distribution histogram of the global rhythmically expressed genes from each plant species (Fig. R1). We found that the expression phase of rhythmically expressed genes clustered in two temporal windows, which are around the light–dark (ZT12, Fig. R1b–f) or dark–light (ZT0, Fig. R1c–e) transition points for the plant species with artificially well-controlled conditions. These results indicate that the rhythmically expressed genes are enriched in morning phase in majority of the examined plant species. Comparing to the artificially experimental conditions, the expression phases with the largest number of rhythmically expressed genes lag approximately 2 hours in rice (similar as ZT2, Fig. R1a) under natural environments. As light is an important environmental stimulus, we speculate that under artificially construct experimental conditions, the rhythmically expressed genes in physiological readiness to the coming light/dark, while the rhythmically expressed genes elicit to expression after light induction/erasure under natural environments. We also checked the gene number in the morning and evening phase, and found that there was no significant difference between morning and evening under artificially controlled conditions. However, the rhythmic genes detected in morning phase (2,337) is more than the rhythmic genes in evening phase (1,479) in rice under fluctuating field conditions. We speculate that the light might be one of stimulus that results in increased number of rhythmic genes in the morning compared to artificially well-controlled conditions.*

4 I don't understand why the dash lines don't locate in the peak of density in Fig. 1f. Is it an error? *Response: The dashed lines indicate the average phase of RNAPII occupancy or mRNA accumulation. The average phase values don't locate in the peak of density plot due to the asymmetry of data distribution. It is not an error, similar plots are also presents in these studies (Koike et al. 2012; Gaidatzis et al. 2015; Takahashi 2017).*

5 The comma is used to separate groups of thousands in some numbers (line 133, 20,667), but not in whole manuscript (line 134, 4526). It is better to use a consistent format in whole manuscript.

*Response: We thank the reviewer for the suggestion. Following the reviewer’s suggestion, we have made revisions throughout the paragraphs to keep consistent format.*

6 Line 297-298, the statistic analysis should be used to check whether the difference is significant. *Response: We thank the reviewer’s suggestion and added the statistic test in the revised Figure S11e and 11f, indicating that the difference is significant. We reanalyzed the data and found that the rhythmic node genes tended to show wider expression breadth than non-rhythmic node genes, which is same as our previous conclusion. However, the length of rhythmic node genes is longer (not shorter) than non-rhythmic node genes, which is opposite to our previous result due to data sorting error. We have revised the conclusion and added the significance test in the newly added Figure S11e and 11f in the revised manuscript. Meanwhile, other conclusions are not affected. We apologize for this mistake.*

Reviewer #2:

This study investigated how RNAPII dynamically interacts with their corresponding regulatory regions during the circadian cycle. Long-read chromatin interaction analysis (ChIA-PET) was used to analyze genome-wide chromatin interactions associated with RNAPII in rice. The interplay between 3D genome architecture, rhythmic RNAPII occupancy, and rhythmic gene expression was also analyzed. The generated RNAPII-associated high resolution dynamic chromatin interactome maps provides new insight for understanding orchestrated circadian transcriptional outputs. The experiments in this study is carefully designed and well conducted. I have the following comments.

Comments:

1. RNAPII signals increased sharply at transcription start sites (TSS), decreased on entering gene body regions, and peaked at polyadenylation site (PAS) (page5, line 106). Please explain why RNAPII signals peaked at polyadenylation site.

*Response: We thank the reviewer for pointing out this and allow us to have chance to explain more about it. The distribution of RNAPII signals peaked at polyadenylation site (PAS) in this study has actually also been observed from 58 of RNAPII ChIP-seq data using unphosphorylated RNAPII antibodies in our previous publication (Zhao et al. 2020). To examine if the phosphorylation of Ser 2P or Ser 5P at the carboxyl-terminal domain (CTD) of RNAPII influence the RNAPII binding pattern, we also generated ChIP-seq data with Ser 2P and Ser 5P CTD-specific RNAPII antibody, respectively (Fig. R2a). We found Ser 2PRNAPII signals increased sharply at transcription start sites (TSS), slowed rising rate on entering gene body regions, and peaked at PAS. These trends were also present in Ser 5P RNAPII occupancy pattern, although the rising rate on entering gene body regions were less pronounced. Furthermore, we analyzed the occupancy profile of the unphosphorylated RNAPII antibodies in human cells (Fig. R2b) and found that the unphosphorylated RNAPII is recruited to the TSS and in a low density of gene body regions. Moreover, unphosphorylated RNAPII density ratio downstream of the PAS is slightly higher than gene body regions, which are distinct of the occupancy patterns observed in rice, but in line with previously reported results in human cells (Li et al. 2012). Together, we speculate that the more abundant/pausing in the vicinity of PAS of RNAPII indicate a potential role of PAS regions in transcriptional regulation in rice.*

2. In total, we identified 20,667 high-confidence (FDR < 0.05) RNAPII-associated chromatin interactions connecting 4526 genes at 08:00 (Fig. 3a) and 21,001 loops connecting 5298 genes at 20:00 (Additional file 1: Figure S6e) (Page 6, line 132).

Please indicate how much overlap between the RNAPII-associated chromatin interactions connecting 4526 genes at 08:00 and those 5298 genes at 20:00.

*Response: We thank the reviewer’s comments. We captured 20,667 RNAPII-associated chromatin interactions at 08:00 and 21,001 interactions at 20:00, and there are 3,316 (9%) of the chromatin interactions were overlapped between two datasets (Fig. 3i). Of 4,526 and 5,298 looping genes at 08:00 and 20:00, 3,033 (45%) looping genes were overlapped between two time points.*

3. OsLHY, a well-known core circadian clock gene in rice [38], was found to form loops with 74 genomic regions at 08:00 in the RNAPII-mediated chromatin connectivity maps (Fig. 2e, f) (Page 6, line 142). The authors may want to describe in more details about those74 genomic regions associated with OsLHY (eg., there are any specific loci that maybe functional associated with OsLHY). In addition to OsLHY, I would like to see the interacting profiles of other core circadian clock genes such as OsTOC1, OsPRR73, and OsELF3. Do they share similar interacting profiles?

*Response: We thank the reviewer for the suggestions. We have examined the details of the genomic regions associated with OsLHY in the RNAPII-mediated chromatin connectivity maps and provided all information in supplementary tables for reference (Additional file 2: Table S8). We also describe the properties of OsLHY-associated chromatin loop connecting genes in the RNAPII-mediated chromatin connectivity maps in the revised manuscript (lines 183-191, Additional file 1: Figure S6m– o).*

*In brief, OsLHY was found to be interacted with 74 genomic regions at 08:00 in the RNAPIImediated chromatin connectivity maps. Of all captured OsLHY looping genes, 46% (33), 44% (31), and 10% (7) were associated with rhythmically, non-rhythmically, and non-expressed genes, respectively. The expression peak phases of OsLHY-associated rhythmically expressed genes were largely (70%, 23 out of 33) clustered in the 06:00–10:00 temporal window (Additional file 1: Figure S6m, n). The expression levels of RNAPII-mediated OsLHY looping rhythmic genes were positively correlated with OsLHY mRNA expression (Pearson rank correlation = 0.71), while others (nonrhythmically and non-expressed genes) were negatively correlated with OsLHY mRNA expression (Pearson rank correlation = -0.38) (Additional file 1: Figure S6o). AmiGO analysis showed that no significantly over-represented Gene Ontology (GO) term enrichment of all the OsLHY looping genes; this may be partly due to the limited number of input genes.*

*Following the reviewer’s suggestions, we also provided the detailed information of the genomic regions associated with OsTOC1, OsPRR37, OsPRR73, OsGI, OsELF3, and OsZTL1 in the RNAPIImediated chromatin connectivity maps at 08:00 and 20:00 datasets, in supplementary tables (Additional file 2: Table S8 and S10). The visualization of chromatin connectivity was constructed of one-hop interactions mediated by core circadian genes at 08:00 and 20:00 was shown in the Additional file 1: Figure S11a, b.*

*To investigate whether the core circadian clock genes share similar interacting profiles, we analyzed the properties of core circadian clock genes-associated looping genes in the RNAPIImediated chromatin connectivity maps. As shown in Fig. R3a, core circadian clock genes-associate gene pairs tended to have the same or close diurnal phase. The expression levels of core circadian clock genes-connected rhythmic genes were positively correlated with core circadian clock genes mRNA expression (Pearson rank correlation = 0.72). while other looping genes (non-rhythmically and non-expressed genes) were negatively correlated with core circadian clock genes mRNA expression (Pearson rank correlation = -0.4) in RNAPII-mediated chromatin networks (Fig. R3b). Motif characterization suggests three regulatory elements (GTGGGNCCCAC, CACGCGC, and CACGTGA), which are TCP family transcription factor (TF), FAR-RED ELONGATED HYPOCOTYLS 3 (FHY3) and its homolog FAR-RED IMPAIRED RESPONSE 1 (FAR1), and bHLHrelated TF binding sites, are highly enriched, respectively (Fig. R3c). These results suggest that TFassociated elements cluster together and may generate nuclear hubs supporting coordinated rhythmic transcription in the nucleus.*

4. In contrast, these RNAPII tethered chromatin loops were virtually undetectable at 20:00, which was the time point of trough OsLHY expression (Fig. 2e). About 34% (569/1631) of jmj705-upregulated and 27% (282/1055) of jmj705 downregulated genes overlapped with those detected in ossnrk1a1 under starvation (Fig 2C) (Page 6, line 145). It appears that there are still some RNAPII tethered chromatin loops at 20:00. Please indicate clearly whether the RNAPII tethered chromatin loops at 20:00 are completely different from those at 8:00.

*Response: There are 20,667 RNAPII tethered chromatin loops at 08:00 and 21,001 loops at 20:00, 91% of these loops between two datasets are different. Only 9% of chromatin loops are overlapped between two time points. We further provided the heatmap showing the time-specific chromatin loops, sorted by the number of paired-end tag (PET) counts (Fig. 3j) and reformulated the conclusion as "In contrast, these RNAPII tethered chromatin loops were virtually undetectable at 20:00 at OsLHY locus, which was the time point of trough OsLHY expression (Fig. 2e)" for better clarity. Please find the details of this part at lines 146-148. It seems that the contents related to jmj705 and ossnrk1a1 were not mentioned in our paper.*

5. Among the 13,738 and 16,983 RNAPII-binding sites detected at 08:00 and 20:00, respectively, 4 6090 (44%) and 6883 (41%) served as chromatin interaction anchors, respectively (Page 7, line 153). Please indicate how much overlap among the 13,738 and 16,983 RNAPII-binding sites detected at 08:00 and 20:00.

*Response: We thank the reviewer for the comments. Of 13,738 RNAPII-binding sites detected at 08:00, there are 8,455 peaks overlapped with 16,983 peaks detected at 20:00. Furthermore, of 6,090 (44%) interaction anchors detected at 08:00 and 6,883 (41%) anchors detected at 20:00, we found 3,608 RNAPII anchor were overlapped. We have added this part to lines 155-158 in the revised manuscript.*

6. Rhythmically expressed node genes within static CSCs with expression peaks were relatively randomly distributed throughout the day and had similar expression levels in the morning and evening (Fig. 4c, d, Additional file 1: Figure S8a) (Page 9, line 217). In Fig. 4c, it appears to me that the rhythmically expressed node genes within static CSCs showed higher levels of expression in the morning than in the evening. Please explain.

*Response: Thank you for pointing out this. We performed the statistical analysis (Student’s t test) to test if the expression level of rhythmically expressed node genes within static CSCs in the morning and evening is significant difference, the result suggests this difference is not significant (p value = 0.66, Fig. R4).*

7. Further investigation of the core node genes in the core circadian gene-associated network revealed 297 that the rhythmic core node genes tended to show a wide expression breadth and short gene length (Fig. 5c) (Page 12, line 295). Please explain what is the meaning of "a wide expression breadth and short gene length".

*Response: We apologize for the confusion due to our unclear description in the main text. The gene expression breadth is defined as the number of tissues in which a transcript is detected. We reanalyzed the data and found that the rhythmic node genes tended to show wider expression breadth than non-rhythmic node genes, which is same as our previous conclusion. However, the length of rhythmic node genes is longer (not shorter) than non-rhythmic node genes, which is opposite to our previous result due to data sorting error. We have revised the conclusion and added the significance test in the newly added Fig S11e and 11f in the revised manuscript. Meanwhile, other conclusions are not affected. We apologize for this mistake.*

8. Moreover, the common node genes among the core circadian clock genes associated with the AM and PM interactome maps showed higher degrees than those in the AM- or PM-specific connectivity maps (Fig. 5d, e) (Page 13, line 298). Please explain what is the meaning of "showed higher degrees".

*Response: The degree of an anchor gene means the overall interaction or connection frequency with other anchor genes. The higher degree of an anchor gene, the more looping genes it has. As shown in Fig. 5e, we performed the statistical significance of the degrees between the common node genes and AM- or PM-specific node genes, and they did show significant difference. We also reword the conclusion with "Moreover, the common node genes among the core circadian clock genes-associated interaction maps showed higher degrees than those in the AM- or PM-specific connectivity maps (Fig. 5d, e)" (lines 319-321) for better clarity.*

9. The expression of TOC1 is repressed at morning. However, TOC1 appeared in both AM and PM specific groups in Fig 5. In addition, the evening genes ELF3 and GI both appeared in the AM specific group in Fig 5. These results need to be explained.

*Response: We agree with the reviewer that the expression of OsTOC1 is repressed in the morning. However, identification of chromatin interactions depends on the presence of RNAPII peaks. Although they are evening genes, the RNAPII peaks at these gene loci are still observed in the 08:00 and 20:00 datasets (Fig. R5), the presence of RNAPII peaks is suggestive of the possibility of the chromatin loop formation. We have discussed this part in the revised manuscript (lines 323-326).*

*References cited in the Response to Referees*

*Dong Q, Li N, Li X, Yuan Z, Xie D, Wang X, Li J, Yu Y, Wang J, Ding B, Zhang Z, Li C, Bian Y, Zhang A, Wu Y, Liu B, Gong L (2018) Genome-wide Hi-C analysis reveals extensive hierarchical chromatin interactions in rice. Plant J 94 (6):1141-1156. doi:10.1111/tpj.13925.*

*Gaidatzis D, Burger L, Florescu M, Stadler MB (2015) Analysis of intronic and exonic reads in RNA-seq data characterizes transcriptional and post-transcriptional regulation. Nat Biotechnol 33 (7):722-729. doi:10.1038/nbt.3269.*

*Greenham K, Sartor RC, Zorich S, Lou P, Mockler TC, McClung CR (2020) Expansion of the circadian transcriptome in Brassica rapa and genome-wide diversification of paralog expression patterns. Elife 9. doi:10.7554/eLife.58993.*

*Koike N, Yoo SH, Huang HC, Kumar V, Lee C, Kim TK, Takahashi JS (2012) Transcriptional architecture and chromatin landscape of the core circadian clock in mammals. Science 338 (6105):349-354. doi:10.1126/science.1226339.*

*Li G, Ruan X, Auerbach RK, Sandhu KS, Zheng M, Wang P, Poh HM, Goh Y, Lim J, Zhang J, Sim HS, Peh SQ, Mulawadi FH, Ong CT, Orlov YL, Hong S, Zhang Z, Landt S, Raha D, Euskirchen G, Wei CL, Ge W, Wang H, Davis C, Fisher-Aylor KI, Mortazavi A, Gerstein M, Gingeras T, Wold B, Sun Y, Fullwood MJ, Cheung E, Liu E, Sung WK, Snyder M, Ruan Y (2012) Extensive promoter-centered chromatin interactions provide a topological basis for transcription regulation. Cell 148 (1-2):84-98. doi:10.1016/j.cell.2011.12.014.*

*Li M, Cao L, Mwimba M, Zhou Y, Li L, Zhou M, Schnable PS, O'Rourke JA, Dong X, Wang W (2019) Comprehensive mapping of abiotic stress inputs into the soybean circadian clock. Proc Natl Acad Sci U S A 116 (47):23840-23849. doi:10.1073/pnas.1708508116.*

*Li X, Luo OJ, Wang P, Zheng M, Wang D, Piecuch E, Zhu JJ, Tian SZ, Tang Z, Li G, Ruan Y (2017) Long-read ChIA-PET for base-pair-resolution mapping of haplotype-specific chromatin interactions. Nat Protoc 12 (5):899-915. doi:10.1038/nprot.2017.012.*

*Muller LM, Mombaerts L, Pankin A, Davis SJ, Webb AAR, Goncalves J, von Korff M (2020) Differential Effects of Day/Night Cues and the Circadian Clock on the Barley Transcriptome. Plant physiology 183 (2):765-779. doi:10.1104/pp.19.01411.*

*Peng Y, Xiong D, Zhao L, Ouyang W, Wang S, Sun J, Zhang Q, Guan P, Xie L, Li W, Li G, Yan J, Li X (2019) Chromatin interaction maps reveal genetic regulation for quantitative traits in maize. Nat Commun 10 (1):2632. doi:10.1038/s41467-019-10602-5.*

*Romanowski A, Schlaen RG, Perez-Santangelo S, Mancini E, Yanovsky MJ (2020) Global transcriptome analysis reveals circadian control of splicing events in Arabidopsis thaliana. Plant J 103 (2):889-902. doi:10.1111/tpj.14776.*

*Takahashi JS (2017) Transcriptional architecture of the mammalian circadian clock. Nat Rev Genet 18 (3):164- 179. doi:10.1038/nrg.2016.15.*

*Tang Z, Luo OJ, Li X, Zheng M, Zhu JJ, Szalaj P, Trzaskoma P, Magalska A, Wlodarczyk J, Ruszczycki B, Michalski P, Piecuch E, Wang P, Wang D, Tian SZ, Penrad-Mobayed M, Sachs LM, Ruan X, Wei CL, Liu ET, Wilczynski GM, Plewczynski D, Li G, Ruan Y (2015) CTCF-Mediated Human 3D Genome Architecture Reveals Chromatin Topology for Transcription. Cell 163 (7):1611-1627. doi:10.1016/j.cell.2015.11.024.*

*Zhao L, Wang S, Cao Z, Ouyang W, Zhang Q, Xie L, Zheng R, Guo M, Ma M, Hu Z, Sung WK, Zhang Q, Li G, Li X (2019) Chromatin loops associated with active genes and heterochromatin shape rice genome architecture for transcriptional regulation. Nat Commun 10 (1):3640. doi:10.1038/s41467-019-11535-9.*

*Zhao L, Xie L, Zhang Q, Ouyang W, Deng L, Guan P, Ma M, Li Y, Zhang Y, Xiao Q, Zhang J, Li H, Wang S, Man J, Cao Z, Zhang Q, Zhang Q, Li G, Li X (2020) Integrative analysis of reference epigenomes in 20 rice varieties. Nat Commun 11 (1):2658. doi:10.1038/s41467-020-16457-5.*

**Second round of review**

**Reviewer 1**

The revised manuscript has addressed all of my concerns.

Line 59, there is a symbol error between 'cis' and 'elements'.

**Reviewer 2**

The authors have satisfactorily responded to my previous review.

**Authors Response**

**Point-by-point responses to the reviewers’ comments:**

Reviewer #1:

The revised manuscript has addressed all of my concerns.

Line 59, there is a symbol error between 'cis' and 'elements'.

*Response: We have corrected the symbol error between 'cis' and 'elements' as suggested by Referee 2 and addressed other editorial requirements in the revised manuscript.*
